# Characterization of *trh2* Harbouring *Vibrio parahaemolyticus* Strains Isolated in Germany

**DOI:** 10.1371/journal.pone.0118559

**Published:** 2015-03-23

**Authors:** Silke Bechlars, Claudia Jäckel, Susanne Diescher, Doreen A. Wüstenhagen, Stefan Kubick, Ralf Dieckmann, Eckhard Strauch

**Affiliations:** 1 Federal Institute for Risk Assessment, Department of Biological Safety, National Reference Laboratory for Monitoring Bacteriological Contamination of Bivalve Molluscs, Berlin, Germany; 2 Fraunhofer Institute for Cell Therapy and Immunology, Branch Bioanalytics and Bioprocesses (Fraunhofer IZI-BB), Potsdam-Golm, Germany; University of Malaya, MALAYSIA

## Abstract

**Background:**

*Vibrio parahaemolyticus* is a recognized human enteropathogen. Thermostable direct hemolysin (TDH) and TDH-related hemolysin (TRH) as well as the type III secretion system 2 (T3SS2) are considered as major virulence factors. As *tdh* positive strains are not detected in coastal waters of Germany, we focused on the characterization of *trh* positive strains, which were isolated from mussels, seawater and patients in Germany.

**Results:**

Ten *trh* harbouring *V*. *parahaemolyticus* strains from Germany were compared to twenty-one *trh* positive strains from other countries. The complete *trh* sequences revealed clustering into three different types: *trh1* and *trh2* genes and a pseudogene *Ψtrh*. All German isolates possessed alleles of the *trh2* gene. MLST analysis indicated a close relationship to Norwegian isolates suggesting that these strains belong to the autochthonous microflora of Northern Europe seawaters. Strains carrying the pseudogene *Ψtrh* were negative for T3SS2β effector *vopC*. Transcription of *trh* and *vopC* genes was analyzed under different growth conditions. *Trh2* gene expression was not altered by bile while *trh1* genes were inducible. *VopC* could be induced by urea in *trh2* bearing strains. Most *trh1* carrying strains were hemolytic against sheep erythrocytes while all *trh2* positive strains did not show any hemolytic activity. TRH variants were synthesized in a prokaryotic cell-free system and their hemolytic activity was analyzed. TRH1 was active against sheep erythrocytes while TRH2 variants were not active at all.

**Conclusion:**

Our study reveals a high diversity among *trh* positive *V*. *parahaemolyticus* strains. The function of TRH2 hemolysins and the role of the pseudogene *Ψtrh* as pathogenicity factors are questionable. To assess the pathogenic potential of *V*. *parahaemolyticus* strains a differentiation of *trh* variants and the detection of T3SS2β components like *vopC* would improve the *V*. *parahaemolyticus* diagnostics and could lead to a refinement of the risk assessment in food analyses and clinical diagnostics.

## Introduction

*Vibrio parahaemolyticus* is a Gram-negative, halophilic bacterium that is found in estuarine environments worldwide [1,2]. It can cause food-borne gastroenteritis most frequently associated with the consumption of raw or undercooked seafood [3–5]. Pathogenic *V*. *parahaemolyticus* strains are responsible for the majority of seafood-associated infections in the United States [6], many Asian countries [7] and South America [8]. Compared to the Asian continent and the USA, *Vibrio parahaemolyticus* infections are rarely reported in Europe. This may be due to a low incidence of illnesses or may be the result of the lack of epidemiological systems for monitoring *Vibrio*-associated illness or *Vibrio* occurrence in seafood [9]. Nevertheless several sporadic outbreaks in UK and Spain and single clinical cases in other European countries have been reported [9]. The pathogenicity of *V*. *parahaemolyticus* is mainly correlated to the possession of genes encoding the thermostable direct hemolysin (TDH) and/or TDH-related hemolysin (TRH) [10, 11]. Five variants of the *tdh* gene have been found, sharing an identity of 96 to 98% [12]. In contrast, *trh* genes possess a significantly broader nucleotide sequence variation and can be subdivided in two main subgroups (*trh1* and *trh2*) which share 84% identity [13]. Both epidemiological studies and animal-based studies indicate that at least one of the two type three secretion systems (T3SS) also plays a major role in pathogenicity of *V*. *parahaemolyticus* [14–17]. T3SS1, located on chromosome 1, is present in all *V*. *parahaemolyticus* strains and involved in cytotoxicity in eukaryotic cell lines *in vitro* [18, 19]. However, analyses suggest that it plays a relatively minor role in intestinal disease [19,20]. T3SS2, located on chromosome 2, is strongly correlated with the presence of *tdh* and/or *trh* and further subdivided with T3SS2α associated with *tdh* on a pathogenicity island (VpaI-7) and T3SS2β located close to *trh* on a different pathogenicity island [21]. The occurence of environmental strains which lack *tdh/trh* but carry T3SS2 [22] and transposase elements flanking the gene cluster [21, 23] leads to the assumption that the correlation is not 100%. Additionally, *trh* is genetically linked to an urease (*ure)* gene cluster associated with a nickel transportation system. The rare urease-positive phenotype of *V*. *parahaemolyticus* within the species *Vibrio* makes the production of urease a good diagnostic marker for potential pathogenic *trh* positive *V*. *parahaemolyticus* strains [11].

As detection of *tdh* positive *V*. *parahaemolyticus* strains in coastal waters of Germany is extremely rare, we focused on the analysis of *trh* positive German *V*. *parahaemolyticus* strains. In general, *trh* harbouring strains are detected in a range of about 3 to 5% at coastlines of Northern Europe [24–26] with tendancy to rise in coastal areas of France [24,27]. Indigenous German *trh* positive strains were compared to a variety of other *trh* positive strains from different countries. In an effort to assess the genetic relationship among *trh* positive isolates we determined the complete coding region of the *trh* genes of all strains and performed multilocus sequence typing (MLST) analysis. Furthermore, all isolates were screened via multiplex PCR for potentially pathogenic targets present in the genome of pandemic strain RIMD2210633. Hemolytic activity and resistance towards human serum were investigated as a measure of virulence-associated phenotypic traits. Strains were cultivated under different growth conditions to examine possible inductive effects on the expression of hemolysins and the T3SS2β effector gene *vopC* with quantitative reverse transcription PCR.

## Material and Methods

### Bacterial strains

A total of thirty-one *V*. *parahaemolyticus* isolates positive for *trh* using the primers Trh-forward and Trh-reverse [28] were obtained from different sources (clinical, environmental, and food retail) in various countries (Table 1). Ten isolates were indigenous strains from the Baltic and North Sea (seven from seawater and two from mussel primary production) and one strain from a patient after consuming fish from the Baltic Sea. Four more strains from Germany were either from imported seafood (three strains) or travel-associated (Tanzania). Two *tdh* positive strains (RIMD2210633 and ATCC43996) and one *tdh/trh* negative strain (VN-0022) were used as controls. Species identification of all strains were performed by standard biochemical assays and species specific PCR targeting the *toxR* gene.

**Table 1:** List of *V*. *parahaemolyticus* strains used in this study.

Strain	Year of isolation	Sample Origin	Country	Source
VN-0024	2006	Ring Trial	unknown	CEFAS
VN-0028^1^ ^)^	1995	Clinical	Tanzania	BfR
VN-0029	1995	Clinical	Germany	BfR
VN-0030^2^ ^)^	1999	Environmental	Germany	BfR
VN-0038	2007	Clinical	Peru	BfR
VN-0045	1988	Clinical	USA	BfR
VN-0046	1994	Clinical	USA	BfR
VN-0049	1991	Clinical	Thailand	BfR
VN-0050	1994	Clinical	Thailand	BfR
VN-0053	1999	Environmental	Indonesia	BfR
VN-0055	1999	Environmental	USA	BfR
VN-0057	1999	Environmental	USA	BfR
VN-0058	1999	Environmental	USA	BfR
VN-0061	2006	Environmental	Spain	BfR
VN-0070	1995	Seafood, Retail	Vietnam^4^ ^)^	BfR
VN-0077	2001	Clinical	Norway	BfR
VN-0084	2010	Seafood, Retail	UK	BfR
VN-0293^3^ ^)^	2011	Primary production	Germany	LAVES
VN-0295	2012	Seafood, Retail	Denmark^4^ ^)^	BfR
VN-0296	2012	Seafood, Retail	Italy^4^ ^)^	BfR
VN-0393^2^ ^)^	1999	Environmental	Germany	BfR
VN-0394^2^ ^)^	1999	Environmental	Germany	BfR
VN-0395^2^ ^)^	1999	Environmental	Germany	BfR
VN-0396	1950	Seafood, Retail	Japan	BfR
VN-2897^2^ ^)^	2011	Environmental	Germany	AWI
VN-3859	2002	Environmental	UK	CEFAS
VN-3933^2^ ^)^	2011	Environmental	Germany	LAGuS
VN-4016	2010	Environmental	Netherlands	AWI
VN-5189^2^ ^)^	2011	Environmental	Germany	IFH
VN-10300^3^ ^)^	2013	Primary production	Germany	LAVES
Control strains				
VN-0022	2010	Seafood, Retail	Ireland^4^ ^)^	BfR
ATCC43996	1972	Clinical	UK	BfR
RIMD 2210633	1983	Clinical	Japan	BfR

^1)^ Travel associated, isolated in Germany after return

^2)^ Environmental German strains were from seawater.

^3)^ Strains isolated from German mussel primary productions

^4)^ Strains isolated in Germany from imported seafood

AWI: Alfred Wegener Institute, Helgoland, Germany;

BfR: Federal Institute for Risk Assessment, Berlin, Germany;

CEFAS: Centre for Environment, Fisheries and Aquaculture Science, Weymouth, United Kingdom;

IFH: Institute of Food Hygiene, Department of Veterinary Medicine, Freie Universität Berlin, Germany;

LAGuS: State Office for Health and Social Affairs, Rostock, Germany;

LAVES: Lower Saxony State Office for Consumer Protection and Food Safety, Cuxhaven, Germany

### Strain cultivation

Strains were routinely grown on Luria-Bertani (LB) agar (Merck, Darmstadt, Germany) at 37°C overnight and further cultivated in LB medium (Merck, Darmstadt, Germany) containing 0.3 M NaCl at 37°C and 200 rpm shaking. For bile and urea induction experiments bacteria were grown for 2 h in LB medium containing 0.3 M NaCl with or without 0.04% of crude bile (bile bovine, Sigma-Aldrich, Steinheim, Germany) or 0.1% urea (Urea, Roth, Karlsruhe, Germany). For testing urease activity single colonies from the agar plate were transferred on urea-dextrose-agar (0.9% caseinpeptone, 0.1% K_2_HPO_4_, 0.5% glucose, saline solution (358 g/l), 1% bromothymol blue, 1% agar, 1% CO(NH_2_)_2_, pH 6.8) and incubated at 37°C overnight.

### Multilocus sequence typing (MLST)

Genomic DNA was extracted using the RTP Bacteria DNA Kit from STRATEC Molecular, Berlin, Germany. MLST was performed as described by Gonzalez-Escalona *et al*. (2008) [29] detecting partial sequences of seven housekeeping genes from both chromosomes. PCR primers targeted *recA* (recA protein), *dnaE* (DNA polymerase III, alpha subunit), *gyrB* (DNA gyrase, subunit B), *dtdS* (threonine dehydrogenase), *pntA* (transhydrogenase alpha subunit), *pyrC* (dihydroorotase) and *tnaA* (tryptophanase). PCR reactions were carried out using primers and applying conditions detailed on the *V*. *parahaemolyticus* MLST website (http://pubmlst.org/vparahaemolyticus/info/protocol.shtml). PCR products were purified using QIAquick PCR purification Kit (Qiagen, Hilden, Germany). Sequencing reactions were performed commercially at Eurofins MWG Operon, Ebersberg, Germany. Primers were synthesized by Metabion International AG, Planegg/Martinsried, Germany. The obtained sequences were queried against the pubMLST database to determine the allele designations and sequence type (ST) of each isolate. Phylogenetic analysis of MLST sequences was performed using Mega 6.0 software [30]. Minimum evolution (ME) trees for concatenated sequences were constructed using the kimura-2 parameter model to estimate the genetic distances.

### PCR genotyping and *trh* sequencing

PCR reactions were performed using a Mastercycler EP Gradient (Eppendorf, Hamburg, Germany) in a volume of 25 μl with 1 x PCR buffer (2 mM MgCl_2_), 0.2 mM of each dNTP, 0.2 μM of each primer, and 1.5 U of Dream Taq DNA Polymerase (Fermentas, St. Leon-Rot, Germany). The PCR running conditions were: an initial denaturation of 95°C for 3 min, 30 cycles of 94°C for 30 s, 50–68°C for 30 s and 72°C for 1 min, and a final elongation step of 72°C for 5 min. To analyze the distance from *trh* to *ureC*, PCR was performed using Long PCR Enzyme Mix (Thermo Scientific, Schwerte, Germany) according to Park *et al*. [11] with minor modifications. PCR reaction was carried out in a 50 μl volume with 1 x Long PCR buffer with 1.5 mM MgCl_2_, 0.2 mM of each dNTP, 0.5 μM of each primer and 1.5 U Long PCR Enzyme Mix. PCR running conditions were as follows: an initial denaturation of 94°C for 3 min, 30 cycles of 98°C for 20 s, 53°C for 20 s and 68°C for 7 min, and a final elongation step of 68°C for 10 min. The PCR primers, annealing temperatures, target genes and amplicon sizes are shown in Table 2.

**Table 2:** Polymerase chain reaction (PCR) primers, targets and amplification sizes used for genotyping.

Target gene			Amplicon	Anneal.	
(putative function)	Primer	Primer sequence 5’ → 3’	size (bp)	temp. (°C)	Ref.
*trh* (hemolysin)	Trh-forward	GGCTCAAAATGGTTAAGC	251	62	[28]
	Trh-reverse	CATTTCCGCTCTCATATGC			
*tdh* (hemolysin)	Tdh-forward	CCACTACCACTCTCATATGC	425	62	[28]
	Tdh-reverse	CCATCTGTCCCTTTTCCTGC			
*vscP* (T3SS1)	VP1670F	ACCGATTACTCAAGGCGATG	392	60	[67]
	VP1670R	TACGTTGTTGGCGTGATTGT			
*vscC2* (T3SS2α)	VPA1339F	GATTCGCGGAACTCAAGAAG	343	60	[67]
	VPA1339R	CTTGTCCGAGATCAACGTCA			
*vscS2* (T3SS2α)	VPA1335F	ATGTAACGGCGGCTAGCTTA	174	60	[67]
	VPA1335R	CAAACTGTGTCAGTAGCACCA			
*vopB2* (T3SS2α)	VPA1362F	CTGCAGGTATCGCATCTTCA	250	60	[67]
	VPA1362R	TTAGAACCAACCGACGAAGC			
*vopT (*T3SS2α*)*	VPA1327F	TGGCGAAAGAGCCATTAGAT	97	60	[67]
	VPA1327R	TCAACTCCAAATTCGCCTTC			
*vscC2* (T3SS2β)	Beta_vscC2_F	GTACTTTGCTGTCTAACC	1404	60	[67]
	Beta_vscC2_R	CTTACTCTTAACTTCCGACG			
*vscS2* (T3SS2β)	Beta_vscS2_F	TTGATGTTGTTTCGGCTAGC	184	60	[67]
	Beta_vscS2_R	CCACCGCCGAACTCGGCTAACAAG			
*vopB2* (T3SS2β)	Beta_vopB2_F	GAGCCTGTTGCTCTATGGAGCCAGG	942	60	[67]
	Beta_vopB2_R	CGACACAGAACGCAATGCTTGCTCG			
*vopC* (T3SS2β)	Beta_vopC_F	AACCAACTTGCGACTAAATC	594	60	[67]
	Beta_vopC_R	TCCCGACAGTTTTTCTGCAC			
VPaI-1	LVPC0387F	CTAACTCTGCCGATGCTGAC	690	58	[68]
(genomic island)	LVPC0387R	GACCTGCCGTGCCAATAAG			
VPaI-2	LVPC0643F	AGCGTCTTGAGTTACCTAATGC	737	58	[68]
(genomic island)	LVPC0643R	CAGTGGAATAGTGCGAATTGAAC			
VPaI-3	LVPC1083F	GGCTTCTTCGGTTAGTATGTCTC	1010	68	[68]
(genomic island)	LVPC1083R	ATGCTGGCTTCTGATATGTTCTC			
VPaI-4	PVP2137L	CAAGCAATACAACGCAAGGAAC	350	58	[68]
(genomic island)	PVP2137R	GGTGGCAGGTTCAACATATCTC			
VPaI-5	PVP2905F	GCCATCGCCCAGCAAATATAG	400	58	[68]
(genomic island)	PVP2905R	CTCCACAGCCTCATCTACATTG			
VPaI-6	LVPCA1262F	GGACCTAACGGAGAACATTCATC	661	58	[68]
(genomic island)	LVPCA1262R	CATTGGCGGAGCGAATAAGG			
*ureC* (urease)	LVPCRPI11F	TCTACGGCGAAGAGGTCAAG	837	54	[68]
	LVPCRPI11R	TCAACATATCCAAGTGCTCATCC			
*Vp1401* (T6SS)	LVPC1401F	GCACGCCACTGAAGTTCTTG	1057	58	[68]
	LVPC1401R	AACGACAGATTGAGCACTTGAAG			
*Vp1390* (T6SS)	VP1390-F	TACCATCAGAGGATACAACC	262	50	[47]
	VP1390-R	AACAATGAGAACATCAAACA			
*Vp1405* (T6SS)	VP1405-F	AACCCAAGAAATATCCGCCC	282	56	[47]
	VP1405-R	TCACCCATTCAAATACCGCC			
*Vp1409* (T6SS)	VP1409-F	TGTTGCTTTCTATTGCGAC	869	58	[47]
	VP1409-R	CCATAACGACTTTTCTTTC			
*Vp0950* (Biofilm)	VP0950-F	GCCAAACTTCTCAAACAACA	298	50	[47]
	VP0950-R	ATGAAACGCAATTTACCATC			
*Vp0952* (Biofilm)	VP0952-F	TATGATGGTGTTTGGTGC	276	50	[47]
	VP0952-R	TGTTTTTCTGAGCGTTTC			
*Vp0962* (Biofilm)	VP0962-F	GACCAAGACCCAGTGAGA	358	50	[47]
	VP0962-R	GGTAAAGCCAGCAAAGTT			
*Vp0394* (MTase)	MTase-F	GTCTTGTCGAATAGAACTCTGA	683	58	[69]
	MTase-R	TAAGCTCCAAAATCCATACG			
*trh-ureC*	P84-F	CATTTCCGCTCTCATATGC	7945	53	[11]
	URE6-R	ATGCTGGAATGATGTTAGGT			
*VPA140* (capsular	VPA1403-F	GCCAACGAGATTCAAAACCAC	420	58	This study
polysaccharide *cpsA)*	VPA1403-R	GATAGCAAGCAGGATTGGTG			
*hutA* (heme	VPA0882-F	CGTTCATTAACTCTGCTCGC	433	58	This study
transport protein)	VPA0882-R	CCGTTGTAAGACTGCTTTCC			
*mshD* (putative	VP2695-F	GCGTGGTTTTGATGAGAACAG	368	58	This study
MSHA pilin)	VP2695-R	TCAGTGTCAGAGGTTGAGTG			
*hutR* (putative	VPA1466-F	TGAACAAAAGGGCGAAACAG	424	58	This study
TonB system receptor)	VPA1466-R	GCTTTCCGTATCACCAGAAC			
*acfD* (accessory	VPA1376-F	GACGCACAAAACGTTCAAAG	436	56	This study
colonization factor)	VPA1376-R	CCCCTCCATTCCAACTAAATC			

Targets for genotyping were selected from website: VFDB- comparative pathogenomic organization of Vibrio (http://www.mgc.ac.cn/cgi-bin/VFs/comp_graph.cgi?Genus=Vibrio&mode=o). PCR based amplification of the complete *trh* gene was accomplished by using different primer combinations (Table 3). Annealing temperature was 54°C for all primer combinations. For sequencing reactions additional internal *trh* targeting primers were used [28]. Phylogenetic analysis of the coding sequence of *trh* gene was performed as described above.

**Table 3:** Primers used in various combinations for amplification of complete *trh* gene.

Primer (forward)	Primersequence 5’ → 3’	References
VPtrhx1_F	CGCATTTTTTCACCATTTCCC	This study
VPtrhx4_F	GGCCTCGCATTTTTTCACC	This study
Primer (reverse)	Primersequence 5’ → 3’	References
VPtrhx1_R	TTCCCTCGAATTACGCAAC	This study
VPtrhx4_R	ACCTTCTGATTTAGTTCCCTCG	This study
IS-ele-Rev1	GTAATGGCTAAGTCGCTGAAC	This study

Annealing temperature was 54°C, amplicon sizes ranged from 600–700 bp.

### Cultivation and RNA isolation

Strains were grown in 20 ml of LB medium containing 0.3 M NaCl at 37°C and 200 rpm shaking to an OD_600_ 0.6. The cultures containing bacteria at a late logarithmic phase (OD_600_ 0.6) were split into four 5 ml subcultures. All four subcultures were twofold diluted with LB medium (0.3 M NaCl) and exhibited a bacterial concentration of approx. 3 x 10^8^ cfu/ml. To investigate temperature effects on transcription one subculture was incubated at 37°C and one subculture at 20°C. To study the influence of bile and urea at 37°C the other two subcultures were additionally supplemented to a final concentration of either 0.04% bile or 0.1% urea. The supplements had been dissolved and filter-sterilized in LB medium at a concentration of 10%. All four subcultures were incubated for 2 h with 200 rpm shaking at either 37°C or 20°C. After incubation 200–500 μl volume of subculture was mixed with RNA Protect Bacteria Reagent (Qiagen, Hilden, Germany) and proceeded according to the manufacturer`s protocol. Before further processing samples were kept frozen at—80°C. RNA was isolated using RNeasy Minikit (Qiagen, Hilden, Germany) according to the manufacturer`s protocol. The removal of DNA was achieved by using RNase free DNase Set (Qiagen, Hilden, Germany) in a reaction volume of 50 μl (2.5% 10 x DNase buffer, 2% DNase, 15.5% RNase free water and 5% RNA). Reactions were incubated for 1.5 h at 37°C with moderate shaking. After incubation RNA was purified by ethanol precipitation. Briefly, 5 μl of sodium acetate (3 M, pH 5.2) and 150 μl of ice-cold 96% ethanol were added to the samples and incubated overnight at—20°C. RNA was pelleted by centrifugation at 14.000 x g for 30 min at 10°C and washed with 75% ethanol. For the complete removal of ethanol RNA pellets were dried in a speedvac and redissolved in 20 μl of RNase free water. To verify that DNA was completely removed RNA samples were tested in a real-time PCR reaction. RNA was isolated in three separate experiments for each growth condition of each strain.

### Real-time quantitative reverse-transcription PCR of virulence genes (qRT-PCR)

RNA concentration was measured using a NanoDrop spectrophotometer (ND-1000, PEQLAB, Erlangen, Germany) and equally diluted to a concentration of 50 ng/μl RNA before starting synthesis of cDNA. For reverse transcription reaction High-Capacity cDNA Reverse Transcription Kit (Life Technologies, Darmstadt, Germany) was used in a volume of 20 μl with 1 x RT buffer, 1 x dNTP mix, 1 x random primers, 50 U reverse transcriptase and 250 ng RNA. Reverse transcription reaction was performed in a Mastercycler EP Gradient (Eppendorf, Hamburg, Germany) following the guidelines of the manufacturer.

To perform a relative quantification of mRNA of the genes *trh1*, *trh2*, *tdh* and *vopC* we chose the comparative C_T_ (ΔΔC_T_) method with the housekeeping gene *gyrB* as the reference gene and growth at 20°C as a control. Primers and TaqMan probes for specific detection of the target genes were synthesized by Metabion and are shown in Table 4. TaqMan probes were tested in standard curve experiments prior to main experiments. The qRT-PCRs were carried out in ABI PRISM 7500 (Applied Biosystems, Darmstadt, Germany) with 1 x TaqMan Universal Master Mix (with ROX) (Applied Biosystems), 0.25 μM primers, 0.2 μM TaqMan probe and 1 μl cDNA as template. PCR conditions were as follows: after initial denaturation at 95°C for 15 min, a cycle of 95°C for 15 s, 56°C for 60 s and 72°C for 30 s was repeated 40 times. Each sample was measured in triplicate. For data analysis we used ExpressionSuite v1.0.3 software (Life Technologies) which includes the student`s t-test for sample group comparisons. Confidence level was chosen to be 0.95.

**Table 4:** Primers and probes used for qRT-PCR.

Target gene	Primer/probe	Primersequence 5’ → 3’	References
	Vp-gyrB-fw	TGAAGGTTTGACTGCCGTTGT	
*gyrB*	Vp-gyrB-rev	TGGGTTTTCGACCAAGAACTCA	This study
	Vp-gyrB-S2	**TAMRA**—TCACCCATCGCCGATTCAACCGCT–**BHQ-2**	
	Vp-tdh-fw	TCCCTTTTCCTGCCCCC	
*tdh*	Vp-tdh-rev	CGCTGCCATTGTATAGTCTTTATC	This study
	Vp-tdh-S1	**FAM—**TGACATCCTACATGACTGTG**—MGB-NFQ**	
	VP_trh1_fw	AAAAGCGTTCACGGTCAATC	
*trh 1*	VP_trh1_rev	CCAGAAAGAGCAGCCATTGT	[70]
	VP_trh1_S	**CY5**—TCACGACTTCAGGCTCAAAA—**BHQ-2**	
	VP_trh2_fw	CCCCAGTTAAGGCAATTGTG	
*trh 2*	VP_trh2_rev	AGGCGCTTAACCACTTTGAA	[70]
	VP_trh2_S	**HEX**—GGACTATTGGACAAACCGAAC—**BHQ**	
	VP_vopC_fw	AGGTGACGTCAGTGTATTGAAAGG	
*vopC*	VP_vopC_rev	CACAGGTAAATGGCAACTGCTTA	This study
	VP_vopC_S	**Fam—**AAGGGCATTGTTGGTGGAGAGAGCAA—**BHQ**	

### Cell-free protein synthesis of TRH variants and TDH2

TRH1 (VN-0038), TRH2–2 (VN-0293), TRH2–3 (VN-0029) and TDH2 (control) were expressed in a prokaryotic cell-free system. Transcription and subsequent translation were directly initiated from PCR templates generated from chromosomal DNA of the strains. Cell-free synthesized hemolysins were mature proteins without signal peptide and protein tags. Synthesis of hemolysins was performed using the batch-formatted cell-free transcription/translation system based on *E*. *coli* lysate. Primers and PCR conditions are listed in (S1 Table and S2 Table). The detailed procedure of DNA template generation and cell-free expression of toxins is described in Bechlars *et al*. (2013) [31]. Briefly, PCR products were generated in a two-step expression PCR (E-PCR), E-PCR products were added to *E*. *coli* lysates and coupled transcription-translation reactions took place in a 90 min incubation step. Reactions were supplemented with ^14^C- labeled leucine for determining total yield and soluble protein via radioactive analysis in a scintillator. To determine the size and homogeneity of radioactively labeled toxins, all proteins were analyzed by *SDS-PAGE* (sodium dodecyl sulphate-polyacrylamide gel electrophoresis) and visualized using a phosphor-imager (Typhoon Trio+, GE Healthcare, Munich, Germany). The detailed analytical procedures have been described previously [31,32]. All experiments were performed twice.

### *In silico* analysis of TRH variants

For *in silico* analysis amino acid sequences of mature protein of all TRH variants and TDH2 were aligned using Accelrys Gene version 2.5 software (Accelrys Ltd., Cambridge, UK).

Homology modelling was performed using the SWISS Model server (http://swissmodel.expasy.org/interactive). The crystal structure of TDH2 (PDB: 3A57) was used as a template for modelling 3D structure of TRH variants.

### Hemolytic activity of cells, culture supernatants and cell-free synthesized proteins

Hemolytic activity of strains was tested with human blood (blood donation service of the German Red Cross, Berlin-Wannsee, Germany) and sheep blood (Thermo Scientific, Schwerte, Germany). Prior to hemolysis assay strains were cultivated with and without bile extract as described for the transcription analysis. Briefly, strains were grown in LB medium to an OD_600_ 0.6 followed by a split and twofold dilution into two subcultures which both exhibited a bacterial concentration of approx. 3 x 10^8^ cfu/ml. One subculture contained just LB medium (0.3 M NaCl) with bacteria and the other subculture contained LB medium (0.3 M NaCl) supplemented with 0.04% bile and bacteria. Both subcultures were incubated for 2 h with 200 rpm shaking at 37°C.

Hemolysis assay was performed according to Mahoney *et al*. [33] with modifications. After 2 h incubation step 150 μl amounts of cultures were combined with 800 μl of 2% erythrocyte suspension in PBS and cells were pelleted at 7000 x g for 4 min. In parallel 1 ml of cultures were centrifuged at 5000 x g for 10 min at 4°C, culture supernatants were filtered (0.2 μm) and also 150 μl amounts were mixed with 800 μl of 2% erythrocyte suspension. Samples were incubated at 37°C for 4 h with 200 rpm shaking. After incubation cells were centrifuged again at 7000 x g for 4 min and the release of hemoglobin into the supernatant was determined by measuring the absorbance at 540 nm in 700 μl volume. By adding 4% Triton X-100 in LB medium and only LB medium to erythrocyte suspensions the maximum and spontaneous hemoglobin release was determined. All experiments were performed twice.

Hemolytic activity of cell-free synthesized TRH variants and TDH2 was tested with sheep, human and rabbit blood. Prior to hemolysis assay, the toxin concentrations of soluble protein were adjusted to 5 μg/ml with 1 x PBS after centrifugation at 16.000 x g for 10 min. Aliquots of 60 μl soluble protein (approx. 300 ng) were mixed with 60 μl PBS containing 4% of erythrocytes (either sheep or rabbit or human) and incubated for one hour at 37°C as decribed by Bechlars *et al*. (2013) [31]. Cell debris was sedimented by centrifugation at 400 x g for 5 min followed by spectro-photometrical measurement (OD_570_) of the released heme present in the supernatant. Extinction values were set in proportion to the maximum loss of heme in the positive control (4% Triton X).

### Serum resistance

The serum resistance test was carried out as described previously [34,35]. Isolates able to grow in the presence of 60–80% human serum were classified as serum resistant, while growth in the presence of 20–40% and 0–10% were designated as intermediate and sensitive, respectively. All strains were tested in triplicate. *E*. *coli* strain K-12 with the plasmid pKT107 carrying a serum resistance determinant served as a positive control, while *E*. *coli* strain K-12 without pKT plasmid served as a negative control.

## Results

### Sequence analysis of complete *trh* genes

**Fig 1:**
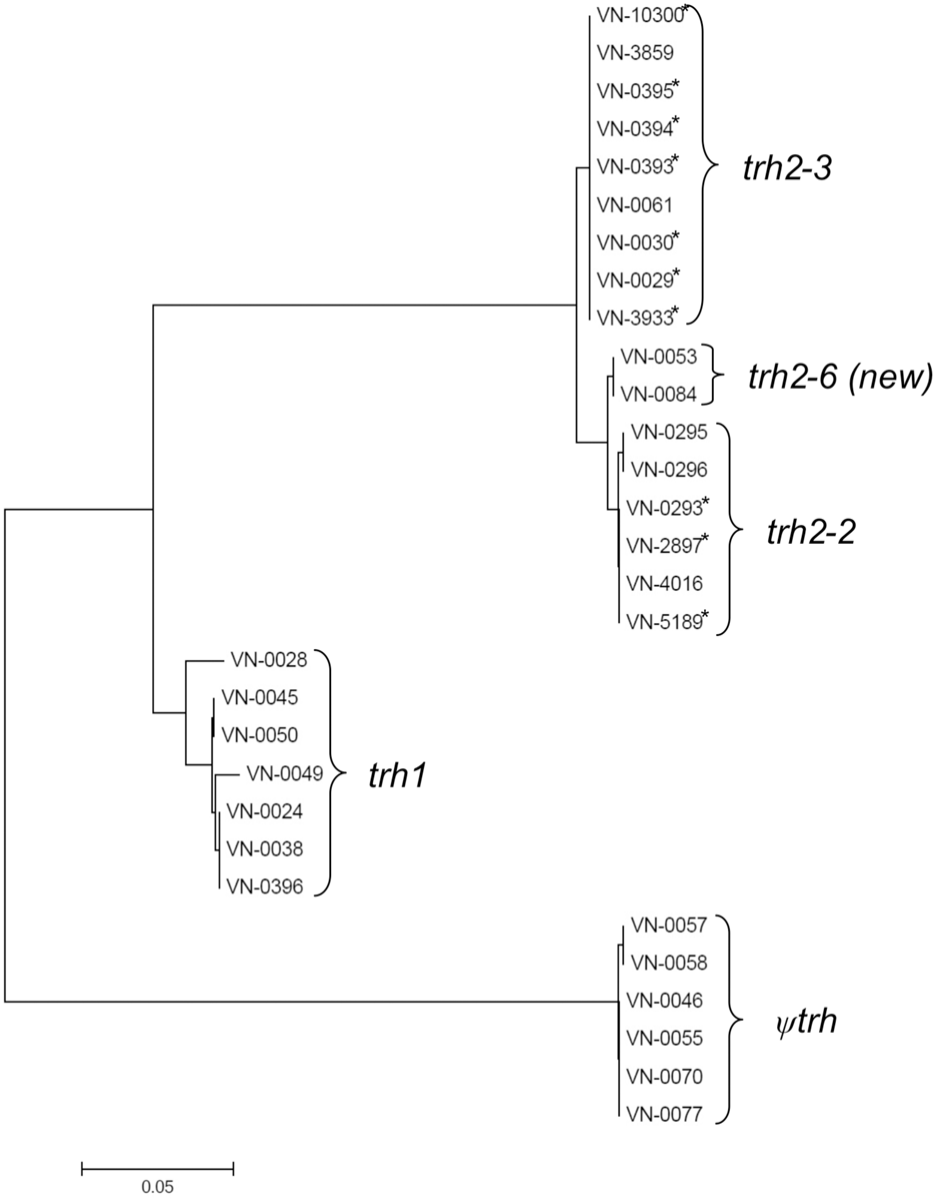
Minimum evolution tree (ME) constructed from coding nucleotide sequences of *trh* gene variants. German strains are indicated by an asterisk.

Thirty-one *V*. *parahaemolyticus* strains (see Table 1) had been identified as *trh* positive by using the primers Trh-forward and Trh-reverse [28] which amplify an internal 251 bp sequence of *trh1/trh2* genes. Sequence analysis of the complete *trh* coding sequences (570 bp) of thirty-one *trh* positive isolates revealed a total of ten different sequence types (Fig. 1, Table 5). Phylogenetic analysis (Minimum Evolution Tree, Mega 6.0) of nucleotide sequences were performed and revealed a clustering of *trh1*, *trh2* genes and a third *trh* sequence variant (Fig. 1). This variant has a modified codon (ATA) instead of an ATG start codon and an early stop codon due to a deletion at position 382 bp. We assume that this sequence is a pseudogene (designated *ψtrh*) which does not encode a functional protein.

**Table 5:** Results of genotyping of *trh* positive strains (see Table 1).

Strain	ST	Hemo-	lysin	T3SS1		T3SS2α
		*tdh*	*trh*	*vscp*	*vscC2*	*vscS2*
VN-0029*	73	-	2	+	-	-
VN-0030*	73	-	2	+	-	-
VN-0293*	79	-	2	+	-	-
VN-0393*	73	-	2	+	-	-
VN-0394*	6	-	2	+	-	-
VN-0395*	73	-	2	+	-	-
VN-2897*	79	-	2	+	-	-
VN-3933*	73	-	2	+	-	-
VN-5189*	79	-	2	+	-	-
VN-0053	967	-	2	+	-	-
VN-0061	64	-	2	+	-	-
VN-0084	985	-	2	+	-	-
VN-0295	uk	-	2	+	-	-
VN-0296	uk	-	2	+	-	-
VN-3859	73	-	2	+	-	-
VN-4016	987	-	2	+	-	-
VN-0028	966	-	1	+	-	-
VN-0049	91	-	1	+	-	-
VN-0396	1	-	1	+	-	-
VN-0024	50	+	1	+	-	-
VN-0038	64	+	1	+	-	-
VN-0045	36	+	1	+	-	-
VN-0050	83	+	1	+	-	-
VN-0046	34	+	ψ	+	-	-
VN-0055	35	+	ψ	+	-	-
VN-0057	26	+	ψ	+	-	-
VN-0058	26	+	ψ	+	-	-
VN-0077	34	+	ψ	+	-	-
VN-0070	452	-	ψ	+	-	-
Control**	3	+	-	+	+	+

Strain	ST		T3SS2β			VpaI-1	VpaI-2
		*vscC2*	*vscS2*	*vopB2*	*vopC*		
VN-0029*	73	+	+	+	+	-	-
VN-0030*	73	+	+	+	+	-	-
VN-0293*	79	+	+	+	+	-	-
VN-0393*	73	+	+	+	+	-	-
VN-0394*	6	+	+	+	+	-	-
VN-0395*	73	+	+	+	+	-	-
VN-2897*	79	+	+	+	+	-	-
VN-3933*	73	+	+	+	+	-	-
VN-5189*	79	+	+	+	+	-	-
VN-0053	967	+	+	+	+	-	-
VN-0061	64	+	+	+	+	-	-
VN-0084	985	+	+	+	+	-	-
VN-0295	uk	+	+	+	+	-	-
VN-0296	uk	+	+	+	+	-	-
VN-3859	73	+	+	+	+	-	-
VN-4016	987	+	+	+	+	-	+
VN-0028	966	+	+	+	+	-	+
VN-0049	91	+	+	+	+	-	-
VN-0396	1	+	+	+	+	-	-
VN-0024	50	+	+	+	+	-	-
VN-0038	64	+	+	+	+	-	-
VN-0045	36	+	+	+	+	-	-
VN-0050	83	+	+	+	+	-	-
VN-0046	34	+	+	+	-	-	+
VN-0055	35	+	+	+	-	-	+
VN-0057	26	+	+	+	-	-	-
VN-0058	26	+	+	+	-	-	-
VN-0077	34	+	+	+	-	-	+
VN-0070	452	-	-	-	-	-	-
Control**	3	-	-	-	-	+	+

Strain	ST	VpaI-3	VpaI-4	VpaI-5	VpaI-6		T6SS		
						*Vp1390*	*Vp1401*	*Vp1405*	*Vp1409*
VN-0029*	73	-	-	-	-	-	+	-	+
VN-0030*	73	-	-	-	-	-	+	-	+
VN-0293*	79	-	-	-	-	-	-	-	+
VN-0393*	73	-	-	-	-	-	+	-	+
VN-0394*	6	-	-	-	-	-	-	-	-
VN-0395*	73	-	-	-	-	-	+	-	+
VN-2897*	79	-	-	-	-	-	-	-	+
VN-3933*	73	-	-	-	-	-	+	-	+
VN-5189*	79	-	-	-	-	-	-	-	+
VN-0053	967	-	-	-	-	-	-	-	-
VN-0061	64	-	-	-	-	-	+	-	+
VN-0084	985	-	-	-	-	-	+	-	+
VN-0295	uk	-	-	-	-	-	-	-	-
VN-0296	uk	-	-	-	-	-	-	-	-
VN-3859	73	-	-	-	-	-	+	-	+
VN-4016	987	-	-	-	-	-	+	-	-
VN-0028	966	-	-	-	-	-	+	-	+
VN-0049	91	-	-	-	-	-	+	-	-
VN-0396	1	-	-	-	-	-	+	-	+
VN-0024	50	-	-	-	-	-	+	-	+
VN-0038	64	-	-	-	-	-	+	-	+
VN-0045	36	-	-	-	-	-	+	-	-
VN-0050	83	-	-	-	-	-	+	-	+
VN-0046	34	-	-	-	-	-	+	-	+
VN-0055	35	-	-	-	-	-	+	-	+
VN-0057	26	-	-	-	-	-	-	-	-
VN-0058	26	-	-	-	-	-	-	-	-
VN-0077	34	-	-	-	-	-	+	-	+
VN-0070	452	-	-	-	-	-	+	-	-
Control**	3	+	+	+	+	+	+	+	+

Strain	ST		Biofilm		MTase	*cpsA*	*mshD*
		*Vp0950*	*Vp0952*	*Vp0962*	*Vp0394*	*VPA1403*	*Vp2695*
VN-0029*	73	+	+	+	-	+	-
VN-0030*	73	+	+	+	-	+	-
VN-0293*	79	+	+	+	-	+	-
VN-0393*	73	+	+	+	-	+	-
VN-0394*	6	+	+	+	-	+	+
VN-0395*	73	+	+	+	-	+	-
VN-2897*	79	+	+	+	-	+	-
VN-3933*	73	+	+	+	-	+	-
VN-5189*	79	+	+	+	-	+	-
VN-0053	967	+	+	+	-	+	-
VN-0061	64	+	+	+	-	+	-
VN-0084	985	+	+	+	-	+	-
VN-0295	uk	+	+	+	-	+	-
VN-0296	uk	+	+	+	-	+	-
VN-3859	73	+	+	+	-	+	-
VN-4016	987	+	+	+	-	+	-
VN-0028	966	+	+	+	-	+	-
VN-0049	91	+	+	+	-	+	+
VN-0396	1	+	+	+	-	+	-
VN-0024	50	+	+	+	-	+	-
VN-0038	64	+	+	+	-	+	-
VN-0045	36	+	+	+	-	+	-
VN-0050	83	+	+	+	-	+	-
VN-0046	34	+	+	+	-	+	+
VN-0055	35	+	+	+	-	+	+
VN-0057	26	+	+	+	-	+	+
VN-0058	26	+	+	+	-	+	+
VN-0077	34	+	+	+	-	+	+
VN-0070	452	+	+	+	-	+	-
Control**	3	+	+	+	+	+	+

Strain	ST	Heme	receptors	*acfD*	*ureC*	*trh-ureC*
		*hutA*	*hutR*	*VPA1376*	*RPI11*	
VN-0029*	73	+	+	-	+	+
VN-0030*	73	+	+	-	+	+
VN-0293*	79	+	+	-	+	+
VN-0393*	73	+	+	-	+	+
VN-0394*	6	+	+	-	+	+
VN-0395*	73	+	+	-	+	+
VN-2897*	79	+	+	-	+	+
VN-3933*	73	+	+	-	+	+
VN-5189*	79	+	+	-	+	+
VN-0053	967	+	+	-	+	+
VN-0061	64	+	+	-	+	+
VN-0084	985	+	+	-	+	+
VN-0295	uk	+	+	-	+	+
VN-0296	uk	+	+	-	+	+
VN-3859	73	+	+	-	+	+
VN-4016	987	+	+	-	+	+
VN-0028	966	+	+	-	+	+
VN-0049	91	+	+	-	+	+
VN-0396	1	+	+	-	+	+
VN-0024	50	+	+	-	+	+
VN-0038	64	+	+	-	+	+
VN-0045	36	+	+	-	+	+
VN-0050	83	+	+	-	+	+
VN-0046	34	+	+	-	+	+
VN-0055	35	+	+	-	+	+
VN-0057	26	+	+	-	+	+
VN-0058	26	+	+	-	+	+
VN-0077	34	+	+	-	+	+
VN-0070	452	+	+	-	-	-
Control**	3	+	+	+	-	-

* German strains are indicated by an asterisk.

**Control strain was the sequenced pandemic strain RIMD2210633 which carries two *tdh* genes and no *trh* gene.

The ten isolates from Germany consisted of seven environmental strains from seawater, two strains from mussel primary production, and one from a patient (VN-0029) and carried alleles of the *trh2* gene. *Trh2* genes of German strains could be divided in *trh2–2* and *trh2–3* adopting the nomenclature from Ellingsen *et al*. [36]. It should be remarked that *trh* sequences published by Ellingsen *et al*. are not complete and are missing 72 bp at the end of sequence. A new *trh2* variant (VN-0053 and VN-0084) which has not been described yet was named *trh2–6*. This new allele encoded a protein that differed in up to 30 amino acid residues from TRH1 variants, in three amino acids from TRH2–3 and in only one amino acid from TRH2–2.

In total six strains of our collection contained a pseudogene *ψtrh*. Five of these strains additionally possessed a *tdh* gene while one strain (VN-0070) only had the pseudogene.

### Multilocus sequence typing

Twenty-nine *trh* positive strains were successfully subtyped at seven loci using multilocus sequence typing (MLST) as described by Gonzalez-Escalona *et al*. [29] and allele numbers and sequence types were assigned according to the PubMLST database. Sequence data were concatenated in the order of loci used to define the allelic profile to produce a single sequence of 3682 bp for each strain.

**Fig 2:**
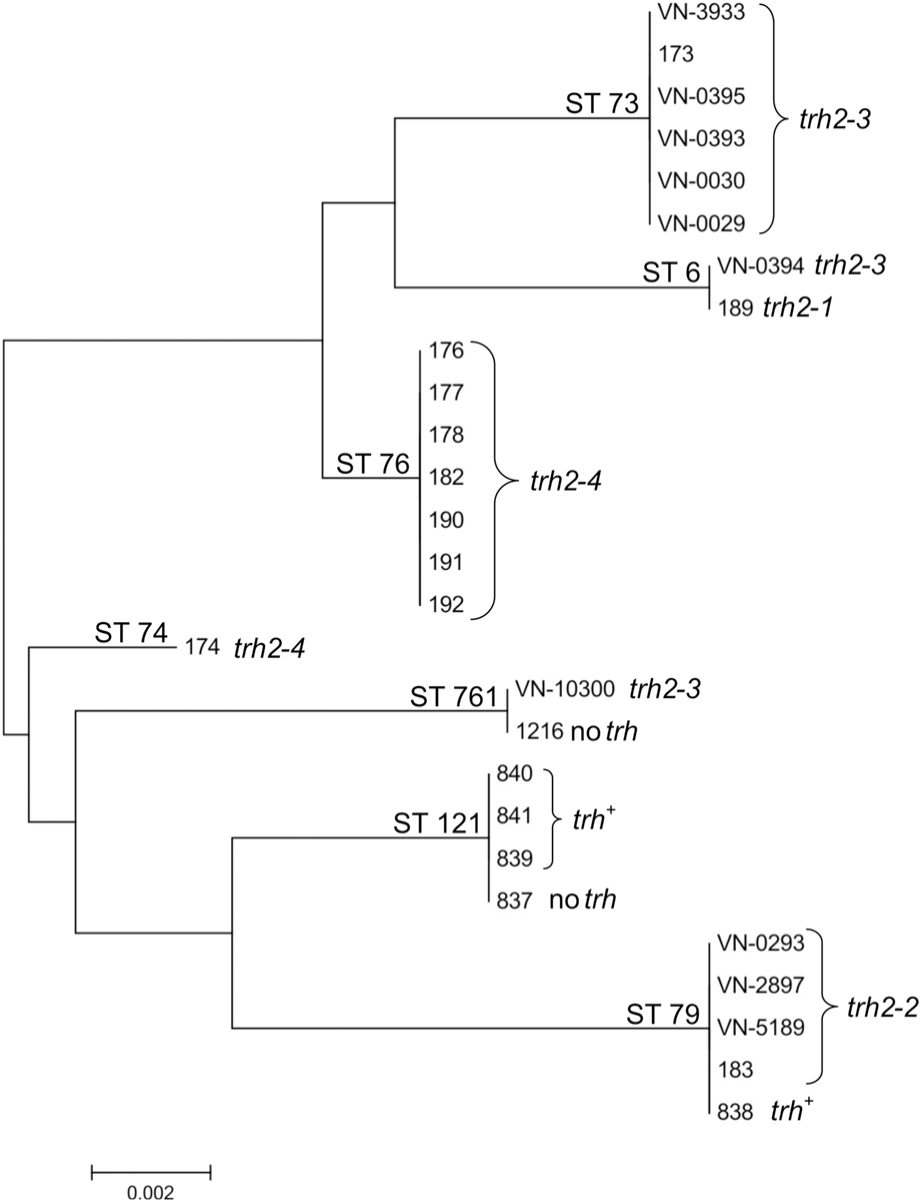
Minimum evolution tree (ME) constructed from the concatenated sequences of seven loci (MLST). Sequence types (STs) of German strains (VN-No.) were compared to *trh* positive strains with identical or related STs of Norwegian and German strains deposited in the pubmlst database (strain-No according to database). All strains present in the database are from Norway or Germany. For some strains no information about *trh* sequence is available (marked as *trh* ^*+*^ *)*. Two strains do not possess a *trh* gene.

Amplification of *recA* gene was difficult in some cases as previously described by Ellingsen *et al*. [36]. For strains VN-0295 and VN-0296 which were from imported retail seafood we could not obtain a complete *recA* sequence. These two strains are therefore not included in the MLST analysis. The main focus of the MLST analysis was to establish the genetic relationships among the ten *trh* positive *V*. *parahaemolyticus* strains isolated from German sources. Results of the phylogenetic analysis based on concatenated MLST sequences are illustrated in a Minimum Evolution Tree shown in Fig. 2. The ten strains were differentiated into four different sequence types (STs), all of which were already present in the MLST database. All isolates carrying *trh2–2* possessed ST79, while most isolates with *trh2–3* had ST73, except for VN-0394 (ST6) and VN-10300 (ST761). Fig. 2 also depicts information of *trh* sequences of other strains of these STs (see discussion, *trh* ^*+*^ indicates sequences with no detailed sequence information).

STs of all remaining *trh* positive *V*. *parahaemolyticus* strains of this study are given in in Table 5. In total four new sequence types were found (strains VN-0028, VN-0053, VN-0084, and VN-4016). Detailed information of the allelic profiles are depicted in S3 Table.

### Genotyping of virulence-associated traits

The presence of a number of virulence-associated genes and putative accessory virulence-associated factors which are present in the genome of pandemic RIMD2210633 strain or were published for *trh* positive strains was tested via multiplex PCR (Table 5). Reference strain RIMD2210633 which carries *tdh* genes was included as a control. Of the 31 *trh* positive strains nine additionally possessed *tdh* sequences (positive in diagnostic PCR using primers Tdh-forward and Tdh-reverse, Table 2). Testing for the presence of T3SS1 and T3SS2 (α and β) revealed that T3SS1 was present in all strains. While T3SS2α was not detected in any of the strains (except for RIMD2210633), three components of T3SS2β (*vscC2*, *vscS2*, and *vopB2*) were present in all *trh* positive strains with the exception of strain VN-0070 which harboured *Ψtrh*. *VopC* was identified in almost all strains, except for the *Ψtrh* carrying strains. Furthermore, we studied the strains for the presence of genomic islands (VpaI-1 to VpaI-6). VpaI-1, VpaI-3, VpaI-4, VpaI-5, and VpaI-6 could not be detected in any strain, while gene *vp0643* of VpaI-2 was present in five strains (VN-0028, VN-0046, VN-0055, VN-077, and VN-4016). The distribution of type six secretion system (T6SS) genes showed a diverse pattern. While genes *vp1390* and *vp1405* were not detected in any of the tested strains, *vp1401* was found in 20 strains and *vp1409* in 19 strains. The following putative accessory virulence-associated genes could be identified in all strains: *vp0950*, *vp0952*, and *vp0962* associated with biofilm formation, capsular polysaccharide biosynthesis glycosyltranferase gene *cpsA* (*vp1403*), *hutA* and *hutR* encoding heme receptors, and *ure* (urease) and *trh-ure* cluster. The coupling of *trh* sequences to the urea biosynthesis cluster (trh-ure) was only missing in one strain (VN-0070) which harboured *Ψtrh* but lacked a *tdh* gene. The gene encoding MTase and the *acfD* gene encoding an accessory colonization factor could not be detected in any of the strains except for the control strain RIMD2210633. *MshD* gene encoding a putative MSHA pilin protein, was detected in four of five *Ψtrh* carrying strains while only two of the remaining strains had *mshD* gene.

### Quantification of virulence gene expression by qRT-PCR

Expression of virulence genes *trh1*, *trh2*, and *tdh*, as well as *vopC* was measured in relation to the *gyrB* gene as a constitutively expressed housekeeping gene [37]. The expression pattern of the genes at 20°C was set as reference and compared to the expression pattern at 37°C with and without addition of bile extract or urea. For the analyses fourteen strains were selected: Eight *trh2* strains (four *trh2–2* and four *trh2–3* strains, seven from German sources), two *trh1* strains, two strains with *trh1* and *tdh* genes, one strain with the combination *Ψtrh* and *tdh*, and one strain with *Ψtrh* only.

**Fig 3:**
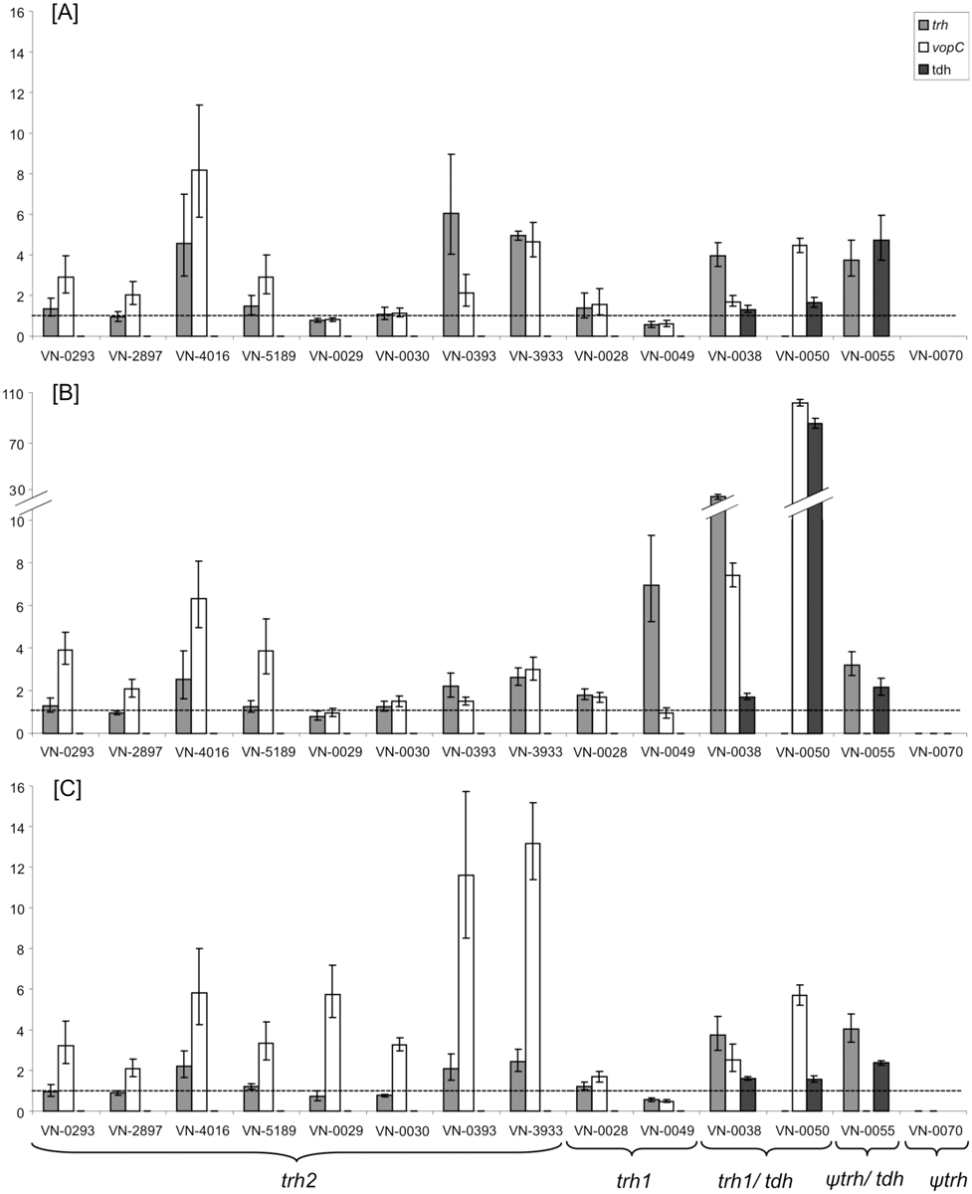
Variation of gene expression of *V*. *parahaemolyticus* strains under different growth conditions. Bacteria were grown at 20°C in LB medium (control, dashed line) and at 37°C with the following conditions: [A] LB medium, [B] LB medium containing 0.04% crude bile and [C] LB medium containing 0.1% urea. The expression of the virulence genes *trh1*, *trh2*, *vopC* and *tdh* and housekeeping gene *gyrB* was determined via qRT-PCR. Using comparative C_T_ (ΔΔC_T_) method for relative quantification (RQ), expression of virulence genes was normalized to the expression of *gyrB*. Means and standard deviations are derived from three separate experiments for each culture condition of each strain.

Transcription analyses showed that strains exhibited diverse transcription patterns of the studied virulence factors and responded differently to the tested growth conditions (see Fig. 3). *Trh2* transcription was slightly increased (approximately 4 to 6 fold) at 37°C compared to 20°C in some strains (VN-0393, VN-3933, and VN-4016), but no influence of bile nor urea on *trh2* expression was observed.

*Trh1* transcription was induced in the presence of bile in strains VN-0038 (30 fold) and VN-0049 (ca. eight fold), while the other two *trh1* positive strains showed no induction of transcription (VN-0028) or no transcription at all (VN-0050). Urea did not have any influence on *trh1* transcription.

*Tdh* transcription of strain VN-0050 was strongly induced (80 fold) in the presence of bile, while no induction of expression was observed for the two other *tdh* positive strains (VN-0038 and VN-0055). Urea had no effect on *tdh* transcription. *Ψtrh* showed no transcription at all (VN-0070) or low transcription rate (VN-0055).

Induced transcription of *vopC*, an effector of T3SS2β, in the presence of bile was observed for *tdh/trh1* positive strains (VN-0038 and VN-0050). While an induced transcription of *vopC* in the presence of urea was detected in strains harbouring *trh2–3* variant (VN-0029, VN-0030, VN-0393, and VN-3933), strains harbouring the *trh2–2* gene showed almost no difference of transcription levels at the three growth conditions at 37°C.

### Hemolysis assay and serum resistance

**Fig 4:**
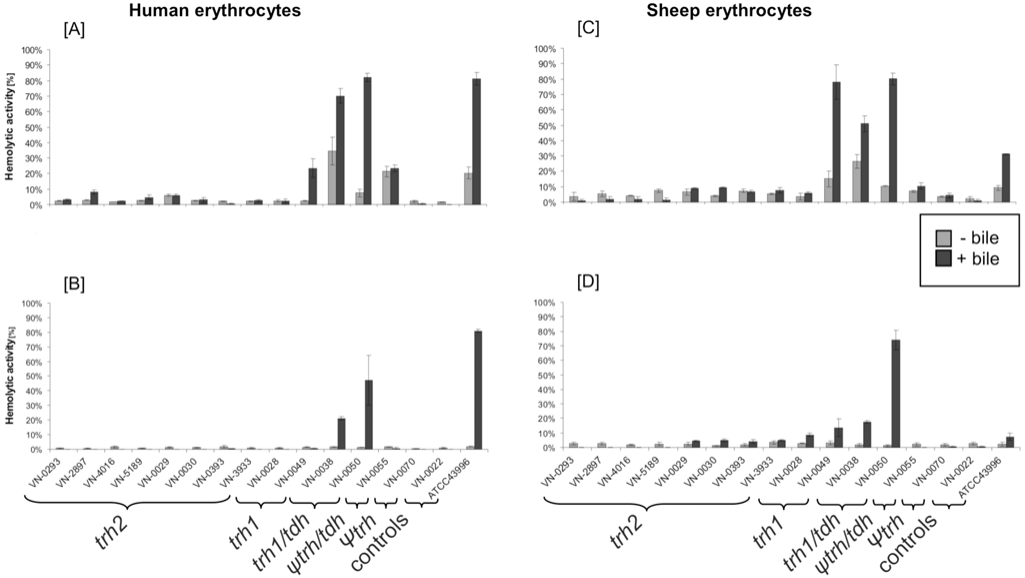
Hemolytic activity of cells and supernatants on human and sheep blood. Bacteria were cultivated in LB medium with and without 0.04% bile bovine. Cultures [A] and [C] and filtered supernatants [B] and [D] were added to a suspension of each blood type and incubated for four hours. Red blood cell lysis was determined at OD_540_. Control strains were VN-0022 (*tdh/trh* negative) and ATCC439996 (*tdh* positive, *trh* negative). All experiments were performed twice.

To verify the expression results of *trh* and *tdh* genes, hemolytic activity of cultures was tested against human and sheep erythrocytes (Fig. 4). Prior to hemolysis assay strains were cultivated with and without bile at 37°C. Additionally, cultures and supernatants were tested separately. We observed no hemolytic activity against both types of erythrocytes for all *trh2* carrying strains. The two *trh1* strains behaved differently; while strain VN-0049 showed strong hemolytic activity against sheep erythrocytes and moderate hemolytic activity on human erythrocytes in response to the induction of bile, VN-0028 exhibited no or very low hemolytic activity. *Trh1/tdh* positive strains revealed strong hemolytic activity in response to bile against both blood types. Relatively low hemolysis was caused by *ψtrh/tdh* (VN-0055) strain and *ψtrh* strain (VN-0070) showed no hemolysis at all. Generally, observed hemolytic activity was always stronger when the bacteria were incubated with erythrocytes than in samples with only culture supernatants added to the erythrocytes.

All tested strains were classified as resistant towards human serum since they were able to grow in the presence of 60–80% serum (data not shown). Only pandemic strain RIMD2210633 was serum sensitive under the tested conditions.

### Cell-free synthesis of TRH variants and hemolytic activity on erythrocytes

Three TRH variants and TDH2 as a control were synthesized in a prokaryotic *in vitro* transcription-translation system with lysates from *E*. *coli*. All PCR products with the expected sizes were produced with similar efficiency (see S1 Fig.). By incorporation of ^14^C-labeled leucine into toxins the rate of protein synthesis was determined. Yields of soluble protein of TRH variants ranged from 12 μg/ml (VN-0029 and VN-0038) to 56 μg/ml (VN-0293), while protein yields of TDH2 were higher (150 μg/ml). Aliquots of the translation mixtures (TMs) and supernatants (SNs) were analyzed for homogeneity and size using SDS-PAGE followed by autoradiography (see S2 Fig.).

Results of hemolytic activity of cell-free expressed toxins on three types of erythrocytes (sheep, human, and rabbit) are summarized in Table 6. Under our test conditions the TRH1 variant (VN-0038) showed hemolytic activity against sheep erythrocytes but not against human and rabbit erythrocytes. Both of our tested TRH2 variants did not show any hemolytic acitivity against all three blood types. TDH2 revealed a stronger hemolytic activity against rabbit erythrocytes (= 50%) than human erythrocytes (= 10%) which is in agreement with Honda *et al*. (1988) [38].

**Table 6:** Hemolytic activity of TRH variants and TDH2 expressed in a cell-free system.

Toxins	Sheep	Human	Rabbit
mTRH1 (VN-0038)	>80%	<0.01%	<0.01%
mTRH2–3 (VN-0029)	<0.01%	<0.01%	<0.01%
mTRH2–2 (VN-0293)	<0.01%	<0.01%	<0.01%
mTDH2 (control)	<0.01%	= 10%	= 50%

Approx. 300 ng of soluble protein were combined with 4% erythrocyte suspension of each blood type respectively. Hemolytic activity was determined by spectro-photometrical measurement at OD_570_ and set in proportion (%) to the maximum hemolysis in the positive control (100%).

m = mature protein (without signal peptide)

## Discussion

### *Trh* sequences and MLST

Sequence analysis of the complete coding regions of all *trh* positive *V*. *parahaemolyticus* strains revealed a clustering into *trh1* and *trh2* genes and a pseudogene *ψtrh*. All strains from German coastal waters possessed alleles of the *trh2* gene and belonged to four STs (ST 73, ST 6, ST 761 and ST 79). These STs were already described in the pubMLST database [36] and included Norwegian and German strains which were all *trh* positive with one exception. Only ST 6 contained a strain isolated outside of Northern Europe (Chile) that lacked a *trh* sequence (not included in Fig. 2). Given the time period of isolation and the geographical distribution the phylogenetic analysis indicates that the *trh2* positive strains are persistent in northern European waters and probably belong to the autochthonous microflora of this region.

In the ME tree (Fig. 2) published *trh2* variants of Norway [36] and some more German isolates with *trh* genes [39] are displayed. Few *trh* negative strains with ST 121 and ST 761 were incorporated into the ME tree. Presence or absence of *trh* genes in strains with the same ST could indicate that these virulence factors may be acquired through horizontal gene transfer, as has been shown for *tdh* genes [40].

Some strains analyzed in our study carried a *trh* pseudogene (*ψtrh*). The frequent occurrence of *trh* pseudogenes has recently been described by Nishibuchi [41]. It should be noted that most of the isolates of our study were *tdh* positive and did not originate from Germany. We assume that *ψtrh* pseudogenes do not encode functional proteins.

Interestingly, *V*. *parahaemolyticus* infections in Europe have been mostly associated with *tdh* positive strains so far and not *trh* [9] except for three clinical cases in Norway [36] and two clinical isolates of our study. Strain VN-0028 which harboured the *trh1* gene, was obtained from a patient after returning from travel to Tanzania. VN-0029 exhibited a *trh2–3* gene and was isolated from a 70-year old patient showing symptoms of diarrhea who had been eating fish from the Baltic Sea. Interestingly, one clinical isolate from Norway (strain 173, Fig. 2) also exhibited the *trh2–3* gene and shared the same ST as VN-0029. In other parts of the world the detection of *trh* in clinical isolates is dominant with 22% (*trh* only) and 59% (*trh/tdh*) in Canada [42], 8.3% (*trh* only) and 24.3% (*trh/tdh*) in North America [43], and 8% (*trh* only) and 34% (*trh/tdh*) in Thailand [44]. In the cited publications, there was no differentiation between *trh1* and *trh2*.

### Genotyping of virulence-associated traits

PCR analysis of *trh* positive *V*. *parahaemolyticus* strains confirmed the genetic linkage of *trh* sequences to the urease gene cluster which has already been noted by others [11, 36, 45]. Only strain VN-0070 with pseudogene *ψtrh* was shown to lack the urea gene cluster and urease activity. VN-0070 did not possess the T3SS2β, which was found by PCR in all other *trh* and *trh/tdh* positive strains. The strains were negative for T3SS2α as has been described already [21].

Interestingly, five strains which were *tdh* positive and possessed the pseudogene *ψtrh*, were negative for the *vopC* gene which encodes an effector protein of T3SS2β, but possessed three other tested proteins of T3SS2β (Table 5). This finding suggests that in the five strains virulence might be attenuated due to an incomplete set of effector genes of the T3SS2β. Based on the observation that the *vopC* gene could be detected only in strains with intact *trh* genes we chose this gene for expression analysis.

Genotyping of further putative virulence-associated traits did not hint to a specific pattern for the *trh* positive strains. Studies showed that genomic islands VpaI-1 comprising MTase gene (Vp0394), VpaI-3, VpaI-4, and VpaI-5 were unique to post-1995 pandemic *V*. *parahaemolyticus* strains [46]. This was confirmed by our finding that no *trh* harbouring strain was positive for these VpaIs and also VpaI-6. Gene *vp0643* of VpaI-2 could be detected in five *trh* positive strains. The presence of some VpaI-2 genes in pathogenic but also non-pathogenic *V*. *parahaemolyticus* strains has been described by Chao *et al*. [47]. The type six secretion system (T6SS) was suggested to be involved in adhesion to host cells and helps to compete against other bacterial species in the environment therefore promoting fitness [46,48]. Chao *et al*. [47] reported that most pandemic strains have the complete set of T6SS genes. Our tested strains harboured a partial set of T6SS genes as it was described for most non-pathogenic strains. The ability to grow in biofilms is a survival strategy used by many bacteria causing infectious diseases [49]. All strains were positive for the tested genes associated with biofilm formation. When analyzing *trh* positive strains for new defined targets (selected from: http://www.mgc.ac.cn/cgi-bin/VFs/comp_graph.cgi?Genus=Vibrio&mode=o) present in the genome of pandemic strain RIMD2210633 no distinct pattern was observed. In conclusion, the genotyping of all strains did not indicate significant differences between clinical strains and strains with environmental or food origin.

### Transcriptional analysis of *trh*, *tdh*, and *vopC* genes

For expression analysis we compared transcription at 20°C to transcription at 37°C and tested the influence of two components present in the human intestine on transcription at 37°C. Bile is an important factor for the digestion of lipids and is excreted by the liver into the small intestine [50]. Urea is the major nitrogenous waste product and is also produced by the liver. 20–25% of all urea produced enters the intestinal tract [51]. In addition, urea is used in agriculture and constitutes >50% of global nitrogenous fertilizer usage. The high input of urea into coastal waters [52] could have an impact on *Vibrio* populations. Urease of *V*. *parahaemolyticus* is expressed and active when urea is present [11]. As in other enteropathogens protective effects of urease were demonstrated [51] and because of the proximate location of the *ure* gene cluster to *trh* and T3SS2β genes in the genome of *V*. *parahaemolyticus* [11], we hypothesized that urea might have an effect on expression of virulence genes.

Our results suggest that the expression of hemolysin genes follows no general pattern. *Trh2* gene expression was slightly increased at 37°C in some strains without the influence of bile and urea (Fig. 3). *Trh1* gene expression could be strongly induced by bile which is to our knowledge the first report of bile-inductive effects on *trh* expression in *V*. *parahaemolyticus*. However, the induction was strain-specific with several strains not responding to bile addition. For *V*. *cholera* (non-O1/non-O139) bile-inductive *trh* expression has already been described [53]. We also observed an increase of *tdh* expression in the presence of bile in some strains which has already been described for pandemic *V*. *parahaemolyticus* strains [54]. Urea addition did not have any significant influence on expression of any of the hemolysin genes.

The expression of the T3SS2β effector gene *vopC* was also analyzed in our transcription analysis. VopC mediates host cell invasion of *V*. *parahaemolyticus* in cultured cells [55] but VopC-mediated invasion is not a required step for pathogenicity in an animal model of infection [56]. Nonetheless, it has been shown that VopC deamidates and therefore activates Rho family GTPases e.g. promoting the formation of actin stress fibres [56]. GTPases play a role in regulation of numerous cellular processes, cell cycle progression and apoptosis [57] and it has been demonstrated that GTPase-mediated signalling is often modulated by various bacterial factors in infection [58]. So, it is possible that VopC combined with other factors takes part in the infection process of *V*. *parahaemolyticus*. Interestingly, we could observe a bile responsive induction of *vopC*, but exclusively for *tdh/trh* bearing strains suggesting a different regulation of the T3SS2β in these strains compared to the remaining strains. The collective triggering of expression of *vopC* with the adjacent located hemolysins in the presence of bile might indicate a mutual regulation of T3SS2β and hemolysins. A bile responsive induction of *tdh* and T3SS2α gene expression of *V*. *parahaemolyticus* has already been demonstrated by Gotoh *et al*. [54]. For a subgroup of *trh2* positive strains—*trh2–3* (VN-0030, VN-0029, VN-0393, VN-3933)—an induction of *vopC* expression in the presence of urea was found. In the other *trh2* positive subgroup of strains—*trh2–2*—*vopC* transcription was slightly increased at 37°C at the three growth conditions suggesting that temperature is a critical factor for triggering *vopC* expression. The upregulation of *vopC* transcription in some strains might contribute to pathogenesis in human infection since it has been demonstrated that T3SS2β is involved in enterotoxicity in animal experiments [21].

An inductive effect of urea on gene transcription is an interesting observation. As a result of overland transport of agriculture fertilizers, urea is also found in coastal waters [52]. Urea in the natural environment of vibrios could have an effect on population structure, as *trh* bearing, urease positive *V*. *parahaemolyticus* strains are unique within the genus *Vibrio* enabling them to use urea as a nitrogen source and could therefore lead to a growth advantage compared to other *Vibrio* strains. An active T3SS2β—although its function in the environment is not known yet- could have an impact on potential predators like protists and increase the environmental fitness as it has been reported for T3SS2α carrying *V*. *parahaemolyticus* strains [59].

### Hemolytic activity on erythrocytes

To verify the transcription studies we tested hemolytic activity of the selected strains against sheep and human erythrocytes with and without bile (Fig. 4). Additionally, we examined the activity of three cell-free synthesized TRH variants against different erythrocyte types (sheep, human, and rabbit). The cell-free protein expression allows the synthesis of active hemolysins from PCR products which can be used directly for testing of hemolytic activity [31].

All *trh2* bearing strains (cultures and supernatants) showed no hemolytic activity against human and sheep erythrocytes, although increased *trh2* transcription was observed in some strains at a temperature of 37°C (VN-0393, VN-3933, and VN-4016). The two cell-free synthesized TRH2 variants also did not show any hemolytic activity towards sheep, human or rabbit erythrocytes. This indicates that lack of hemolytic activity of *trh2* strains is probably due to the missing functionality of the *trh2* gene product and not caused by lack of expression. This is in agreement with the suggestion by Kishishita *et al*. [13] that low or no hemolytic activity is a result of protein functionality and/or binding activity.

The cell-free synthesized TRH1 variant of strain VN-0038 was highly hemolytic against sheep erythrocytes, while under our test conditions no hemolysis of human and rabbit erythrocytes was observed. Similarly, purified TRH1 was shown to be 40 to 50 fold more active against sheep erythrocytes than against human and rabbit erythrocytes [38]. When testing culture and supernatants of this strain, activity against sheep and human erythrocytes was found. This might probably be due to the fact that the strain harboured *tdh* and *trh* genes.

*In silico* analysis of the protein sequences provides one possible interpretation for non-hemolyic activity of the two TRH2 variants since they share very hydrophobic amino acids (aa) at position 156 and 165. TRH forms a tetramer in solution [60]. Performing homology modelling on the basis of TDH structure these two positions (156 and 165) are predicted to be located in the central pore of the tetramer structure which is thought to function as an ion channel. Furthermore, analysis of the tetrameric structure of TDH2 by Yanagihara *et al*. [61] suggested an interaction between the conserved amino acid glutamic acid at position 138 (E138, also conserved in all TRH variants) with the amino acid glutamine at position 165 (Q165), which contributes to the stabilization of the tetrameric structure. The amino acid changes in the two TRH2 variants (as in all TRH2 proteins of this study) could therefore lead to a dysfunction of the hemolysins since it has been demonstrated that maintenance of tetrameric structure with a central pore is indispensable for biological activity of TDH and TRH [60,61]. Noticeably, most TRH1 variants shared the same amino acids at positions 156 and 165 with VN-0038, while the TRH1 variant of strain VN-0028 had the same aa variation as the TRH2 variants and was not hemolytic. While we could not detect hemolytic activity of the TRH2 hemolysins, it cannot be excluded that other biological activities may be present. It has been demonstrated that TRH1 variants show various biological activities including fluid accumulation in rabbit ileal loops, increase of rabbit skin vascular permeability, and cardiotoxicity on cultured myocardial cells [62,63].

When combining the results of the transcription studies with the results obtained from exposing erythrocytes to cultures or supernatants of strains carrying *trh1* or *trh1*/*tdh* or *ψtrh/tdh*, no clear picture arises. Bacterial cells used in hemolysis assays probably express various virulence factors (e.g. T3SS) which could lead to lysis of erythrocytes. However, when only supernatants of cultures were used, the hemolysin activity could be subject to proteolytic activity of extracellular and cell-associated proteases and vary during the growth phase [33].

## Conclusion

*Trh* is considered as a virulence factor since there is a strong association with gastroenteritis [64]. The investigation of all thirty-one *trh* carrying strains for the presence of virulence factors by PCR (Table 5) did not reveal any significant differences between isolates from clinical, environmental, and food sources. Strains originating from Germany only possessed alleles of the *trh2* gene. Although clinical strains carrying *trh2* genes have been found [36, 65] and one German strain (VN-0029) was isolated from a patient that had diarrhea due to consumption of fish from the Baltic Sea, we could not detect hemolytic properties of TRH2 proteins. This may question the role of TRH2 as a virulence factor. However, it cannot be ruled out that other biological activities associated with virulence described for TRH1, like e.g. fluid accumulation in rabbit ileal loops, increase of rabbit skin vascular permeability, and cardiotoxicity on cultured myocardial cells [62], may still be functional in TRH2 proteins. We will address these questions in the future using different human intestinal cells as targets for TRH2 proteins.

Nevertheless, it seems likely that other virulence factors may contribute to infection and illness caused by *V*. *parahemolyticus* strains. This is supported by the reports about *tdh/trh* negative *V*. *parahaemolyticus* strains which caused acute gastroenteritis [66]. It is an interesting finding that all intact *trh* genes were linked to the presence of *vopC*, an effector protein of the T3SS2β, while in strains harbouring a pseudogene (*ψtrh*) *vopC* was not detected. We conclude that a further differentiation of *trh* variants and including the detection of T3SS2β components like *vopC* could be an important step to improve the characterization of potential pathogenic *V*. *parahaemolyticus* and could lead to a refinement of the risk assessment in food analyses and clinical diagnostics.

### Nucleotide sequence accesion number

The *trh* sequences of the following *V*. *parahaemolyticus* strains were deposited at the European Nucleic Archive (http://www.ebi.ac.uk/ena/data/view/accesion number): VN-0028 (Accession#: LM993801), VN-0029 (Accession#: LM993802), VN-0038 (Accession#: LM993803), VN-0049 (Accession#: LM993804), VN-0057 (Accession#: LM993808), VN-0084 (Accession#: LM993807), VN-0295 (Accession#: LM993805) and VN-2897 (Accession#: LM993806).

## Supporting Information

S1 Fig PCR templates for expression of TRH variants and TDH2 generated by E-PCR. An aliquot (1 μl) of E-PCR2 product was analysed on a 1% agarosegel, lane 1: mTRH2 (VN-0029), lane 2: mTRH1 (VN-0038), lane 3: mTRH2 (VN-0293), lane 4: mTDH2 (control), lane 5: no template control reaction (NTC), marker lane: M, mass ladder (50 ng): M1 and mass ladder (100 ng): M2
(TIF)

S2 Fig Synthesis of ^14^C labeled proteins. 5 μl aliquots of radiolabeled cell-free synthesis reactions (translation mixture (TM): 1–5 and supernatants (SN): 6–10) were loaded on a 10% SDS-PAGE gel (up); ^14^C labeled proteins were visualized after electrophoresis with a phosphorimager system (Typhoon TRIO + Imager, GE Healthcare) (down). Lane 1: no template control reaction (NTC) TM, lane 2: mTRH2–3 TM (VN-0029), lane 3: mTRH1 TM (VN-0038), lane 4: mTRH2–2 TM (VN-0293), lane 5: mTDH2 TM (control), marker lane: M, lane 6: NTC SN, lane 7: mTRH2–3 SN (VN-0029), lane 8: mTRH1 SN (VN-0038), lane 9: mTRH2–2 SN (VN-0293) and lane 10: mTDH2 SN (control)
(TIF)

S3 Fig Protein alignment of mature TRH variants and mature TDH2 (165 aa length). Almost all conserved amino acids described for TDH which may participate in π-cation interactions and maintain tetrameric structures (e.g. R46, E138 and Y140) are conserved in all TRH variants. Two possibly relevant amino acid changes were identified. At position 165 TDH2 and TRH1 (VN-0038) possess a hydrophilic aa residue, glutamine (Q) and asparagine (N) respectively, while TRH1 (VN-0028) and both TRH2 variants possess an isoleucine (I) which is regarded as very hydrophobic. At position 156 TDH2 and TRH1 (VN-0038) exhibit hydrophilic/neutral amino acids (glutamic acid, E; serine, S) while TRH1 (VN-0028) and both TRH2 variants again possess a very hydrophobic amino acid (leucine, L). Using SWISS Model server (http://swissmodel.expasy.org/interactive) predicted 3D structure of TRH variants was modelled on the basis of crystal structure of TDH2—which forms a tetramer in solution as TRH [61, 62]. Amino acids at position 165 and 156 of TDH2 are located within and on the edge of the pore formed by the tetramer.
(TIF)

S1 Table A) Primer used for E-PCR1. Bold letters indicate gene specific sequences. Amplified genes have product size of 533 bp. B) PCR conditions for E-PCR1. PCR reactions were carried out in a 50 μl volume as described [31].
(DOCX)

S2 Table Primer used for E-PCR2. Amplified genes have a product size of 728 bp. PCR conditions are described [31].
(DOCX)

S3 Table Sequence types (STs) with allelic profiles of MLST analysis. (DOCX)

## References

[1] Kaneko T, Colwell RR. Incidence of *Vibrio parahaemolyticus* in Chesapeake Bay. Appl Microbiol. 1975; 30: 251–257. 1164012 10.1128/am.30.2.251-257.1975 PMC187162

[2] Nair GB, Ramamurthy T, Bhattacharya SK, Dutta B, Takeda Y, Sack DA, et al Global dissemination of *Vibrio parahaemolyticus* serotype O3:K6 and its serovariants. Clin Microbiol Rev. 2007; 20: 39–48. 17223622 10.1128/CMR.00025-06 PMC1797631

[3] Blake PA, Weaver RE, Hollis DG. Diseases of humans (other than cholera) caused by vibrios. Annu Rev Microbiol. 1980; 34: 341–367. 7002028 10.1146/annurev.mi.34.100180.002013

[4] Thompson FL, Iida T, Swings J. Biodiversity of vibrios. Microbiol Mol Biol Rev. 2004; 68: 403–431. 15353563 10.1128/MMBR.68.3.403-431.2004 PMC515257

[5] Anonymous. Risk assessment of *Vibrio parahaemolyticus* in seafood. World Health Organization, Food and Agriculture Organization of The United Nations. 2011; http://www.fao.org/docrep/014/i2225e/i2225e2200.pdf.

[6] Mead PS, Slutsker L, Dietz V, McCaig LF, Bresee JS, Shapiro C, et al Food-related illness and death in the United States. Emerg Infect Dis. 1999; 5: 607–625. 10511517 10.3201/eid0505.990502 PMC2627714

[7] Pan TM, Chiou CS, Hsu SY, Huang HC, Wang TK, Chiu SI, et al Food-borne disease outbreaks in Taiwan, 1994. J FormosMed Assoc. 1996; 95: 417–420. 8688712

[8] Velazquez-Roman J, Leon-Sicairos N, de Jesus Hernández-Díaz L, Canizalez-Roman A. Pandemic *Vibrio parahaemolyticus* O3:K6 on the American continent. Front Cell Infect Microbiol. 2013; 3: 110 10.3389/fcimb.2013.00110 24427744 PMC3878053

[9] Baker-Austin C, Stockley L, Rangdale R, Martinez-Urtaza J. Environmental occurrence and clinical impact of *Vibrio vulnificus* and *Vibrio parahaemolyticus*: A European perspective. Environ Microbiol Rep. 2010; 2: 7–18. 10.1111/j.1758-2229.2009.00096.x 23765993

[10] Nishibuchi M, Kaper JB. Thermostable direct hemolysin gene of *Vibrio parahaemolyticus*: a virulence gene acquired by a marine bacterium. Infect Immun. 1995; 63: 2093–2099. 7768586 10.1128/iai.63.6.2093-2099.1995 PMC173271

[11] Park KS, Iida T, Yamaichi Y, Oyagi T, Yamamoto K, Honda T. Genetic characterization of DNA region containing the *trh* and *ure* genes of *Vibrio parahaemolyticus* . Infect Immun. 2000; 68: 5742–5748. 10992480 10.1128/iai.68.10.5742-5748.2000 PMC101532

[12] Nishibuchi M, Kaper JB. Duplication and variation of the thermostable direct haemolysin (*tdh*) gene in *Vibrio parahaemolyticus* . Mol Microbiol. 1990; 4: 87–99. 2319944 10.1111/j.1365-2958.1990.tb02017.x

[13] Kishishita M, Matsuoka N, Kumagai K, Yamasaki S, Takeda Y, Nishibuchi M. Sequence variation in the thermostable direct hemolysin-related hemolysin (*trh*) gene of *Vibrio parahaemolyticus* . Appl Environ Microbiol. 1992; 58: 2449–2457. 1514791 10.1128/aem.58.8.2449-2457.1992 PMC195802

[14] Hiyoshi H, Kodama T, Saito K, Gotoh K, Matsuda S, Akeda Y, et al VopV, an F-actin-binding type III secretion effector, is required for *Vibrio parahaemolyticus*-induced enterotoxicity. Cell Host Microbe. 2011; 10: 401–409. 10.1016/j.chom.2011.08.014 22018240

[15] Zhang L, Orth K. Virulence determinants for *Vibrio parahaemolyticus* infection. Curr Opin Microbiol. 2013; 16: 70–77. 10.1016/j.mib.2013.02.002 23433802

[16] Zhou X, Gewurz BE, Ritchie JM, Takasaki K, Greenfeld H, Kieff E, et al A *Vibrio parahaemolyticus* T3SS effector mediates pathogenesis by independently enabling intestinal colonization and inhibiting TAK1 activation. Cell Rep. 2013; 3: 1690–1702. 10.1016/j.celrep.2013.03.039 23623501 PMC3711673

[17] Ritchie JM, Rui H, Zhou X, Iida T, Kodoma T, Ito S, et al Inflammation and disintegration of intestinal villi in an experimental model for *Vibrio parahaemolyticus*-induced diarrhea. PLoS Pathog. 2012; 8. 10.1371/journal.ppat.1002593 PMC3305451 22438811

[18] Kodama T, Rokuda M, Park KS, Cantarelli VV, Matsuda S, Iida T, et al Identification and characterization of VopT, a novel ADP-ribosyltransferase effector protein secreted via the *Vibrio parahaemolyticus* type III secretion system 2. Cell Microbiol. 2007; 9: 2598–2609. 17645751 10.1111/j.1462-5822.2007.00980.x

[19] Hiyoshi H, Kodama T, Iida T, Honda T. Contribution of *Vibrio parahaemolyticus* virulence factors to cytotoxicity, enterotoxicity, and lethality in mice. Infect Immun. 2010; 78: 1772–1780. 10.1128/IAI.01051-09 20086084 PMC2849405

[20] Park KS, Ono T, Rokuda M, Jang MH, Okada K, Iida T, et al Functional characterization of two type III secretion systems of *Vibrio parahaemolyticus* . Infect Immun. 2004; 72: 6659–6665. 15501799 10.1128/IAI.72.11.6659-6665.2004 PMC523034

[21] Okada N, Iida T, Park KS, Goto N, Yasunaga T, Hiyoshi H, et al Identification and characterization of a novel type III secretion system in *trh*-positive *Vibrio parahaemolyticus* strain TH3996 reveal genetic lineage and diversity of pathogenic machinery beyond the species level. Infect Immun. 2009; 77: 904–913. 10.1128/IAI.01184-08 19075025 PMC2632016

[22] Caburlotto G, Gennari M, Ghidini V, Tafi M, Lleo MM. Presence of T3SS2 and other virulence-related genes in *tdh*-negative *Vibrio parahaemolyticus* environmental strains isolated from marine samples in the area of the Venetian Lagoon, Italy. FEMS Microbiol Ecol. 2009; 70: 506–514. 10.1111/j.1574-6941.2009.00764.x 19744242

[23] Chen Y, Stine OC, Badger JH, Gil AI, Nair GB, Nishibuchi M, et al Comparative genomic analysis of *Vibrio parahaemolyticus*: Serotype conversion and virulence. BMC Genomics. 2011; 12. 10.1186/1471-2164-12-294 PMC3130711 21645368

[24] Hervio-Heath D, Colwell RR, Derrien A, Robert-Pillot A, Fournier JM, Pommepuy M. Occurrence of pathogenic vibrios in coastal areas of France. J Appl Microbiol. 2002; 92: 1123–1135. 12010553 10.1046/j.1365-2672.2002.01663.x

[25] Böer SI, Heinemeyer EA, Luden K, Erler R, Gerdts G, Janssen F, et al Temporal and spatial distribution patterns of potentially pathogenic *Vibrio* spp. at recreational beaches of the German north sea. Microb Ecol. 2013; 65: 1052–1067. 10.1007/s00248-013-0221-4 23563708

[26] Ellingsen AB, Jørgensen H, Wagley S, Monshaugen M, Rørvik LM. Genetic diversity among Norwegian *Vibrio parahaemolyticus* . J Appl Microbiol. 2008; 105: 2195–2202. 10.1111/j.1365-2672.2008.03964.x 19120665

[27] Robert-Pillot A, Guenole A, Lesne J, Delesmont R, Fournier JM, Quilici ML. Occurrence of the *tdh* and *trh* genes in *Vibrio parahaemolyticus* isolates from waters and raw shellfish collected in two French coastal areas and from seafood imported into France. Int J Food Microbiol. 2004; 91: 319–325. 14984780 10.1016/j.ijfoodmicro.2003.07.006

[28] Tada J, Ohashi T, Nishimura N, Shirasaki Y, Ozaki H, Fukushima S, et al Detection of the thermostable direct hemolysin gene (*tdh*) and the thermostable direct hemolysin-related hemolysin gene (*trh*) of *Vibrio parahaemolyticus* by polymerase chain reaction. Mol Cell Probes. 1992; 6: 477–487. 1480187 10.1016/0890-8508(92)90044-x

[29] Gonzalez-Escalona N, Martinez-Urtaza J, Romero J, Espejo RT, Jaykus LA, DePaola A. Determination of molecular phylogenetics of *Vibrio parahaemolyticus* strains by multilocus sequence typing. J Bacteriol. 2008; 190: 2831–2840. 10.1128/JB.01808-07 18281404 PMC2293261

[30] Tamura K, Stecher G, Peterson D, Filipski A, Kumar S. MEGA6: Molecular Evolutionary Genetics Analysis version 6.0. Mol Biol Evol. 2013; 30: 2725–2729. 10.1093/molbev/mst197 24132122 PMC3840312

[31] Bechlars S, Wüstenhagen DA, Dragert K, Dieckmann R, Strauch E, Kubick S. Cell-free synthesis of functional thermostable direct hemolysins of *Vibrio parahaemolyticus* . Toxicon 2013; 76: 132–142. 10.1016/j.toxicon.2013.09.012 24060377

[32] Stech M, Merk H, Schenk JA, Stocklein WF, Wüstenhagen DA, Micheel B, et al Production of functional antibody fragments in a vesicle-based eukaryotic cell-free translation system. J Biotechnol. 2012; 164: 220–231. 10.1016/j.jbiotec.2012.08.020 22982167

[33] Mahoney JC, Gerding MJ, Jones SH, Whistler CA. Comparison of the pathogenic potentials of environmental and clinical *Vibrio parahaemolyticus* strains indicates a role for temperature regulation in virulence. Appl Environ Microbiol. 2010; 76: 7459–7465. 10.1128/AEM.01450-10 20889774 PMC2976215

[34] Bier N, Bechlars S, Diescher S, Klein F, Hauk G, Duty O, et al Genotypic diversity and virulence characteristics of clinical and environmental *Vibrio vulnificus* isolates from the baltic sea region. Appl Environ Microbiol 2010; 79: 3570–3581. 10.1128/AEM.00477-13 23542621 PMC3675912

[35] Moll A, Cabello F, Timmis KN. Rapid assay for the determination of bacterial resistance to the lethal activity of serum. FEMS Microbiol Lett. 1979; 6: 273–276.

[36] Ellingsen AB, Olsen JS, Granum PE, Rørvik LM, González-Escalona N. Genetic characterization of trh positive *Vibrio* spp. isolated from Norway. FCIMB 2013; 3: Article 107 10.3389/fcimb.2013.00107 24400227 PMC3872308

[37] Caburlotto G, Lleò MM, Hilton T, Huq A, Colwell RR, Kaper JB. Effect on human cells of environmental Vibrio parahaemolyticus strains carrying type III secretion system 2. Infect Immun. 2010; 78: 3280–3287. 10.1128/IAI.00050-10 20479084 PMC2897368

[38] Honda T, Ni YX, Miwatani T. Purification and characterization of a hemolysin produced by a clinical isolate of Kanagawa phenomenon-negative *Vibrio parahaemolyticus* and related to the thermostable direct hemolysin. Infect Immun. 1988; 56: 961–965. 3126151 10.1128/iai.56.4.961-965.1988 PMC259398

[39] Urmersbach S, Alter T, Koralage MS, Sperling L, Gerdts G, Messelhäusser U, et al Population analysis of *Vibrio parahaemolyticus* originating from different geographical regions demonstrates a high genetic diversity. BMC Microbiol. 2014; 14: 59 10.1186/1471-2180-14-59 24606756 PMC4015679

[40] Kamruzzaman M, Bhoopong P, Vuddhakul V, Nishibuchi M. Detection of a functional insertion sequence responsible for deletion of the thermostable direct hemolysin gene (*tdh*) in *Vibrio parahaemolyticus* . Gene 2008; 421: 67–73. 10.1016/j.gene.2008.06.009 18598741

[41] Nishibuchi M. Recent developments in seafood safety with respect to *Vibrio parahaemolyticus* Abstract booklet conference VIBRIO 2014 Edinburgh, UK pp. 21–22.

[42] Banerjee SK, Kearney AK, Nadon CA, Peterson CL, Tyler K, Bakouche L, et al Phenotypic and genotypic characterization of Canadian clinical isolates of *Vibrio parahaemolyticus*—2000 to 2009. J Clin Microbiol. 2014; 52: 1081–1088. 10.1128/JCM.03047-13 24452166 PMC3993483

[43] Jones JL, Lüdeke CHM, Bowers JC, Garrett N, Fischer M, Parsons MB, et al Biochemical, serological, and virulence characterization of clinical and oyster *Vibrio parahaemolyticus* isolates. J Clin Microbiol. 2012; 50: 2343–2352. 10.1128/JCM.00196-12 22535979 PMC3405591

[44] Thongjun J, Mittraparp-Arthorn P, Yingkajorn M, Kongreung J, Nishibuchi M, Vuddhakul V. The Trend of *Vibrio parahaemolyticus* Infections in Southern Thailand from 2006 to 2010. Trop Med Health 2013; 41: 151–156. 10.2149/tmh.2013-06 24478592 PMC3880868

[45] Iida T, Suthienkul O, Park KS, Tang GQ, Yamamoto RK, Ishibashi M, et al Evidence for genetic linkage between the *ure* and *trh* genes in *Vibrio parahaemolyticus* . J Medl Microbiol. 1997; 46: 639–645. 10.1099/00222615-46-8-639 9511811

[46] Ceccarelli D, Hasan NA, Huq A, Colwell RR. Distribution and dynamics of epidemic and pandemic Vibrio parahaemolyticus virulence factors. FCIMB 2013; 3: Article 97 24377090 10.3389/fcimb.2013.00097 PMC3858888

[47] Chao G, Jiao X, Zhou X, Wang F, Yang Z, Huang J, et al Distribution of genes encoding four pathogenicity islands (VPaIs), T6SS, biofilm, and type i pilus in food and clinical strains of V*ibrio parahaemolyticus* in China. Foodborne Pathog Dis, 2010; 7: 649–658. 10.1089/fpd.2009.0441 20132020

[48] Salomon D, Gonzalez H, Updegraff BL, Orth K. *Vibrio parahaemolyticus* type VI secretion system 1 is activated in marine conditions to target bacteria, and is differentially regulated from system 2. PLoSOne 2013; 8: e61086 10.1371/journal.pone.0061086 23613791 PMC3628861

[49] Enos-Berlage JL, Guvener ZT, Keenan CE, McCarter LL. Genetic determinants of biofilm development of opaque and translucent *Vibrio parahaemolyticus* . Mol Microbiol. 2005; 55: 1160–1182. 15686562 10.1111/j.1365-2958.2004.04453.x

[50] Carey MC, Small DM, Bliss CM. Lipid digestion and absorption. AnnuRev Physiol. 1983; 45: 651–677. 6342528 10.1146/annurev.ph.45.030183.003251

[51] Burne RA, Chen YY. Bacterial ureases in infectious diseases. Microbes Infect. 2000; 2: 533–542. 10865198 10.1016/s1286-4579(00)00312-9

[52] Glibert PM, Harrison J, Heil C, Seitzinger S. Escalating worldwide use of urea—a global change contributing to coastal eutrophication. Biogeochemistry 2006; 77: 441–463.

[53] Miller KA, Hamilton E, Dziejman M. The *Vibrio cholerae trh* gene is coordinately regulated in vitro with type iii secretion system genes by VttrA/VttrB but does not contribute to caco2-bbe cell cytotoxicity. Infect Immun. 2012; 80: 4444–4455. 10.1128/IAI.00832-12 23045478 PMC3497436

[54] Gotoh K, Kodama T, Hiyoshi H, Izutsu K, Park KS, Dryselius R, et al Bile acid-induced virulence gene expression of *Vibrio parahaemolyticus* reveals a novel therapeutic potential for bile acid sequestrants. PLoS ONE 2010; 5: e13365 10.1371/journal.pone.0013365 20967223 PMC2954181

[55] Zhang L, Krachler AM, Broberg CA, Li Y, Mirzael H, Gilpin CJ, et al Type III Effector VopC Mediates Invasion for *Vibrio* Species. Cell Rep. 2012; 1: 453–460. 10.1016/j.celrep.2012.04.004 22787576 PMC3392014

[56] Okada R, Zhou X, Hiyoshi H, Matsuda S, Chen X, Akeda Y, et al The *Vibrio parahaemolyticus* effector VopC mediates Cdc42-dependent invasion of cultured cells but is not required for pathogenicity in an animal model of infection. Cell Microbiol. 2014; 16: 938–947. 10.1111/cmi.12252 24345190 PMC4670550

[57] Lemonnier M, Landraud L, Lemichez E. Rho GTPase-activating bacterial toxins: from bacterial virulence regulation to eukaryotic cell biology. FEMS Microbiol Rev. 2007; 31: 515–534. 17680807 10.1111/j.1574-6976.2007.00078.x

[58] Boquet P, Lemichez E. Bacterial virulence factors targeting Rho GTPases: parasitism or symbiosis? Trends Cell Biol. 2003; 13: 238–246. 12742167 10.1016/s0962-8924(03)00037-0

[59] Matz C, Nouri B, McCarter L, Martinez-Urtaza J. Acquired type III secretion system determines environmental fitness of epidemic *Vibrio parahaemolyticus* in the interaction with bacterivorous protists. PLoS ONE 2011; 6: e20275 10.1371/journal.pone.0020275 21629787 PMC3100340

[60] Ohnishi K, Nakahira K, Unzai S, Mayanagi K, Hashimoto H, Shiraki K, et al Relationship between heat-induced fibrillogenicity and hemolytic activity of thermostable direct hemolysin and a related hemolysin of *Vibrio parahaemolyticus* . FEMS Microbiol Lett. 2011; 318: 10–17. 10.1111/j.1574-6968.2011.02233.x 21291495

[61] Yanagihara I, Nakahira K, Yamane T, Kaieda S, Mayanagi K, Hamada D, et al Structure and functional characterization of *Vibrio parahaemolyticus* thermostable direct hemolysin. J Biol Chem. 2010; 285: 16267–16274. 10.1074/jbc.M109.074526 20335168 PMC2871494

[62] Nelapati S, Nelapati K, Chinnam BK. *Vibrio parahaemolyticus*- An emerging foodborne pathogen-A Review. Vet World 2012; 5: 48–63.

[63] Honda T, Ni YX, Hata A, Yoh MS, Miwatani T, Okamoto T, et al Properties of a hemolysin related to the thermostable direct hemolysin produced by Kanagawa phenomenon negative, clinical isolate of *Vibrio parahemolyticus* . Can J Microbiol. 1990; 36: 395–399. 2118824 10.1139/m90-069

[64] Shirai H, Ito H, Hirayama T, Nakamoto Y, Nakabayashi N, Kumagai K, et al Molecular epidemiologic evidence for association of thermostable direct hemolysin (TDH) and TDH-related hemolysin of *Vibrio parahaemolyticus* with gastroenteritis. Infect Immun. 1990; 58: 3568–3573. 2228229 10.1128/iai.58.11.3568-3573.1990 PMC313699

[65] Okuda J, Ishibashi M, Abbott SL, Janda JM, Nishibuchi M. Analysis of the thermostable direct hemolysin (tdh) gene and the tdh- related hemolysin (trh) genes in urease-positive strains of Vibrio parahaemolyticus isolated on the west coast of the United States. J Clin Microbiol. 1997; 35: 1965–1971. 9230364 10.1128/jcm.35.8.1965-1971.1997 PMC229885

[66] Ottaviani D, Leoni F, Serra R, Serracca L, Decastelli L, Rocchegiani E, et al Nontoxigenic *Vibrio parahaemolyticus* strains causing acute gastroenteritis. J Clin Microbiol. 2012; 50: 4141–4143. 10.1128/JCM.01993-12 23052317 PMC3502970

[67] Noriea NF III, Johnson CN, Griffitt KJ, Grimes DJ. Distribution of type III secretion systems in Vibrio parahaemolyticus from the northern Gulf of Mexico. J Appl Microbiol. 2010; 109: 953–962. 10.1111/j.1365-2672.2010.04722.x 20408916

[68] Xiao X, Yan Y, Zhang Y, Wang L, Liu X, et al A novel genotyping scheme for Vibrio parahaemolyticus with combined use of large variably-presented gene clusters (LVPCs) and variable-number tandem repeats (VNTRs). Intl J Food Microbiol. 2011; 149: 143–151. 10.1016/j.ijfoodmicro.2011.06.014 21742395

[69] Wang HZ, Wong MML, O'Toole D, Mak MMH, Wu RSS, et al Identification of a DNA methyltransferase gene carried on a pathogenicity island-like element (VPAI) in Vibrio parahaemolyticus and its prevalence among clinical and environmental isolates. Appl Env Microbiol. 2006; 72: 4455–4460. 16751568 10.1128/AEM.02095-05 PMC1489626

[70] Messelhäusser U, Colditz J, Thärigen D, Kleih W, Höller C, et al Detection and differentiation of Vibrio spp. in seafood and fish samples with cultural and molecular methods. Int J Food Microbiol. 2010; 142: 360–364. 10.1016/j.ijfoodmicro.2010.07.020 20688407

