# Correction: Characterization of *trh2* Harbouring *Vibrio parahaemolyticus* Strains Isolated in Germany

**DOI:** 10.1371/journal.pone.0126569

**Published:** 2015-04-22

**Authors:** The PLOS ONE Staff

There are errors in Table 5. Two results columns were inadvertently omitted. The publisher apologizes for the error. Please see the corrected Table 5 here.

**Table 5:** Results of genotyping of trh positive strains (see Table 1).

Strain	ST			T3SS1	T3SS2α	T3SS2α	T3SS2α	T3SS2α	T3SS2β	T3SS2β	T3SS2β	T3SS2β	VpaI-1	VpaI-2	VpaI-3	VpaI-4	VpaI-5
		*tdh*	*trh*	*vscp*	*vscC2*	*vscS2*	*vopB2*	*vopT*	*vscC2*	*vscS2*	*vopB2*	*vopC*					
VN-0029*	73	-	2	+	-	-	-	-	+	+	+	+	-	-	-	-	-
VN-0030*	73	-	2	+	-	-	-	-	+	+	+	+	-	-	-	-	-
VN-0293*	79	-	2	+	-	-	-	-	+	+	+	+	-	-	-	-	-
VN-0393*	73	-	2	+	-	-	-	-	+	+	+	+	-	-	-	-	-
VN-0394*	6	-	2	+	-	-	-	-	+	+	+	+	-	-	-	-	-
VN-0395*	73	-	2	+	-	-	-	-	+	+	+	+	-	-	-	-	-
VN-2897*	79	-	2	+	-	-	-	-	+	+	+	+	-	-	-	-	-
VN-3933*	73	-	2	+	-	-	-	-	+	+	+	+	-	-	-	-	-
VN-5189*	79	-	2	+	-	-	-	-	+	+	+	+	-	-	-	-	-
VN-0053	967	-	2	+	-	-	-	-	+	+	+	+	-	-	-	-	-
VN-0061	64	-	2	+	-	-	-	-	+	+	+	+	-	-	-	-	-
VN-0084	985	-	2	+	-	-	-	-	+	+	+	+	-	-	-	-	-
VN-0295	uk	-	2	+	-	-	-	-	+	+	+	+	-	-	-	-	-
VN-0296	uk	-	2	+	-	-	-	-	+	+	+	+	-	-	-	-	-
VN-3859	73	-	2	+	-	-	-	-	+	+	+	+	-	-	-	-	-
VN-4016	987	-	2	+	-	-	-	-	+	+	+	+	-	+	-	-	-
VN-0028	966	-	1	+	-	-	-	-	+	+	+	+	-	+	-	-	-
VN-0049	91	-	1	+	-	-	-	-	+	+	+	+	-	-	-	-	-
VN-0396	1	-	1	+	-	-	-	-	+	+	+	+	-	-	-	-	-
VN-0024	50	+	1	+	-	-	-	-	+	+	+	+	-	-	-	-	-
VN-0038	64	+	1	+	-	-	-	-	+	+	+	+	-	-	-	-	-
VN-0045	36	+	1	+	-	-	-	-	+	+	+	+	-	-	-	-	-
VN-0050	83	+	1	+	-	-	-	-	+	+	+	+	-	-	-	-	-
VN-0046	34	+	ψ	+	-	-	-	-	+	+	+	-	-	+	-	-	-
VN-0055	35	+	ψ	+	-	-	-	-	+	+	+	-	-	+	-	-	-
VN-0057	26	+	ψ	+	-	-	-	-	+	+	+	-	-	-	-	-	-
VN-0058	26	+	ψ	+	-	-	-	-	+	+	+	-	-	-	-	-	-
VN-0077	34	+	ψ	+	-	-	-	-	+	+	+	-	-	+	-	-	-
VN-0070	452	-	ψ	+	-	-	-	-	-	-	-	-	-	-	-	-	-
Control**	3	+	-	+	+	+	+	+	-	-	-	-	+	+	+	+	+

Strain	VpaI-6	T6SS	T6SS	T6SS	T6SS	Biofilm	Biofilm	Biofilm	MTase	*cpsA*	*mshD*	Heme	receptors	*acfD*	*ureC*	*trh-ureC*
		*Vp1390*	*Vp1401*	*Vp1405*	*Vp1409*	*Vp0950*	*Vp0952*	*Vp0962*	*Vp0394*	*VPA1403*	*Vp2695*	*hutA*	*hutR*	*VPA1376*	*RPI11*	
VN-0029*	-	-	+	-	+	+	+	+	-	+	-	+	+	-	+	+
VN-0030*	-	-	+	-	+	+	+	+	-	+	-	+	+	-	+	+
VN-0293*	-	-	-	-	+	+	+	+	-	+	-	+	+	-	+	+
VN-0393*	-	-	+	-	+	+	+	+	-	+	-	+	+	-	+	+
VN-0394*	-	-	-	-	-	+	+	+	-	+	+	+	+	-	+	+
VN-0395*	-	-	+	-	+	+	+	+	-	+	-	+	+	-	+	+
VN-2897*	-	-	-	-	+	+	+	+	-	+	-	+	+	-	+	+
VN-3933*	-	-	+	-	+	+	+	+	-	+	-	+	+	-	+	+
VN-5189*	-	-	-	-	+	+	+	+	-	+	-	+	+	-	+	+
VN-0053	-	-	-	-	-	+	+	+	-	+	-	+	+	-	+	+
VN-0061	-	-	+	-	+	+	+	+	-	+	-	+	+	-	+	+
VN-0084	-	-	+	-	+	+	+	+	-	+	-	+	+	-	+	+
VN-0295	-	-	-	-	-	+	+	+	-	+	-	+	+	-	+	+
VN-0296	-	-	-	-	-	+	+	+	-	+	-	+	+	-	+	+
VN-3859	-	-	+	-	+	+	+	+	-	+	-	+	+	-	+	+
VN-4016	-	-	+	-	-	+	+	+	-	+	-	+	+	-	+	+
VN-0028	-	-	+	-	+	+	+	+	-	+	-	+	+	-	+	+
VN-0049	-	-	+	-	-	+	+	+	-	+	+	+	+	-	+	+
VN-0396	-	-	+	-	+	+	+	+	-	+	-	+	+	-	+	+
VN-0024	-	-	+	-	+	+	+	+	-	+	-	+	+	-	+	+
VN-0038	-	-	+	-	+	+	+	+	-	+	-	+	+	-	+	+
VN-0045	-	-	+	-	-	+	+	+	-	+	-	+	+	-	+	+
VN-0050	-	-	+	-	+	+	+	+	-	+	-	+	+	-	+	+
VN-0046	-	-	+	-	+	+	+	+	-	+	+	+	+	-	+	+
VN-0055	-	-	+	-	+	+	+	+	-	+	+	+	+	-	+	+
VN-0057	-	-	-	-	-	+	+	+	-	+	+	+	+	-	+	+
VN-0058	-	-	-	-	-	+	+	+	-	+	+	+	+	-	+	+
VN-0077	-	-	+	-	+	+	+	+	-	+	+	+	+	-	+	+
VN-0070	-	-	+	-	-	+	+	+	-	+	-	+	+	-	-	-
Control**	+	+	+	+	+	+	+	+	+	+	+	+	+	+	-	-
