# Supplementary figures and images for: Characterization of trh2 Harbouring Vibrio parahaemolyticus Strains Isolated in Germany
Source: PLoS One. 2015 Mar 23;10(3):e0118559. doi: 10.1371/journal.pone.0118559 (PMC4370738; doi:10.1371/journal.pone.0118559)

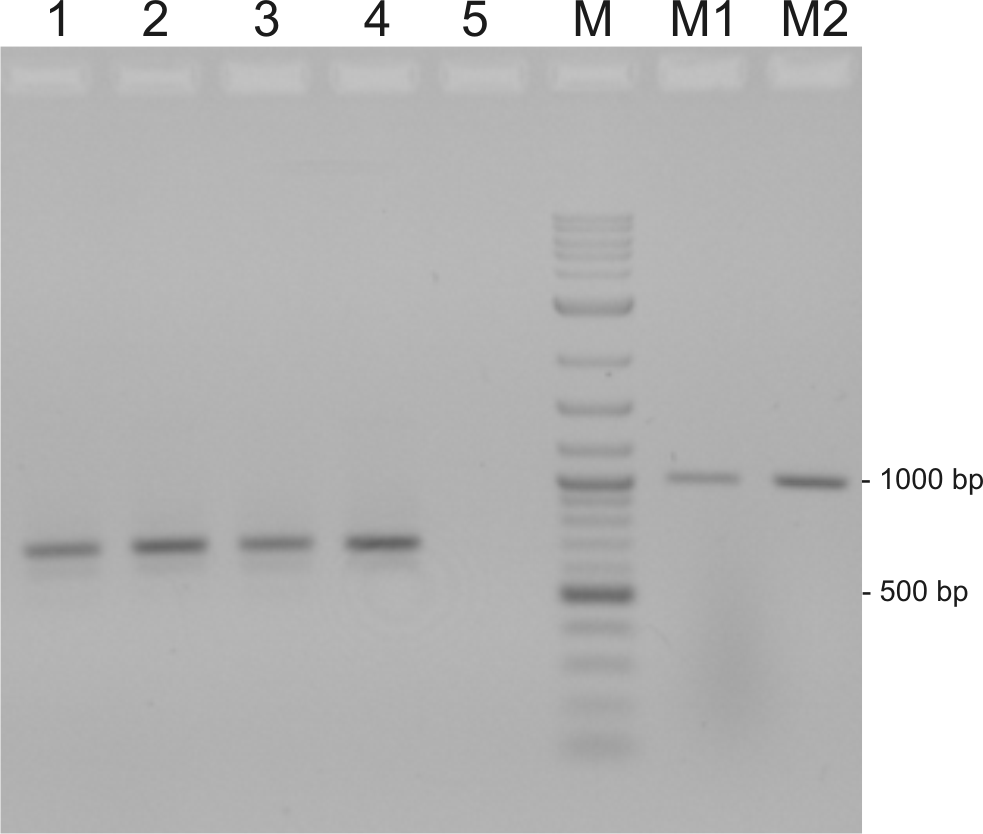

Supplement: S1 Fig — An aliquot (1 μl) of E-PCR2 product was analysed on a 1% agarosegel, lane 1: mTRH2 (VN-0029), lane 2: mTRH1 (VN-0038), lane 3: mTRH2 (VN-0293), lane 4: mTDH2 (control), lane 5: no template control reaction (NTC), marker lane: M, mass ladder (50 ng): M1 and mass ladder (100 ng): M2 (TIF) [file pone.0118559.s001.tif]

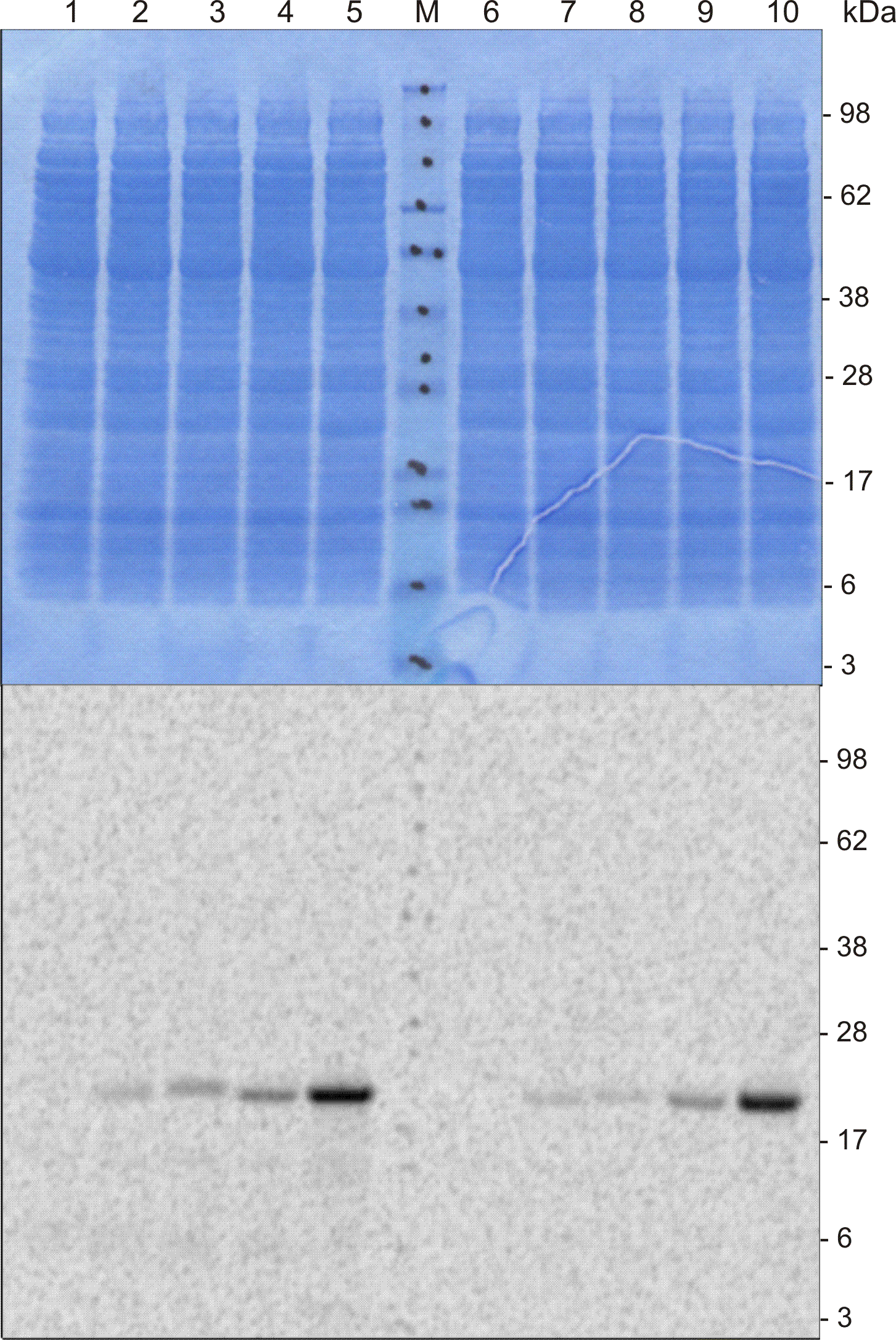

Supplement: S2 Fig — 5 μl aliquots of radiolabeled cell-free synthesis reactions (translation mixture (TM): 1–5 and supernatants (SN): 6–10) were loaded on a 10% SDS-PAGE gel (up); 14C labeled proteins were visualized after electrophoresis with a phosphorimager system (Typhoon TRIO + Imager, GE Healthcare) (down). Lane 1: no template control reaction (NTC) TM, lane 2: mTRH2–3 TM (VN-0029), lane 3: mTRH1 TM (VN-0038), lane 4: mTRH2–2 TM (VN-0293), lane 5: mTDH2 TM (control), marker lane: M, lane 6: NTC SN, lane 7: mTRH2–3 SN (VN-0029), lane 8: mTRH1 SN (VN-0038), lane 9: mTRH2–2 SN (VN-0293) and lane 10: mTDH2 SN (control) (TIF) [file pone.0118559.s002.tif]

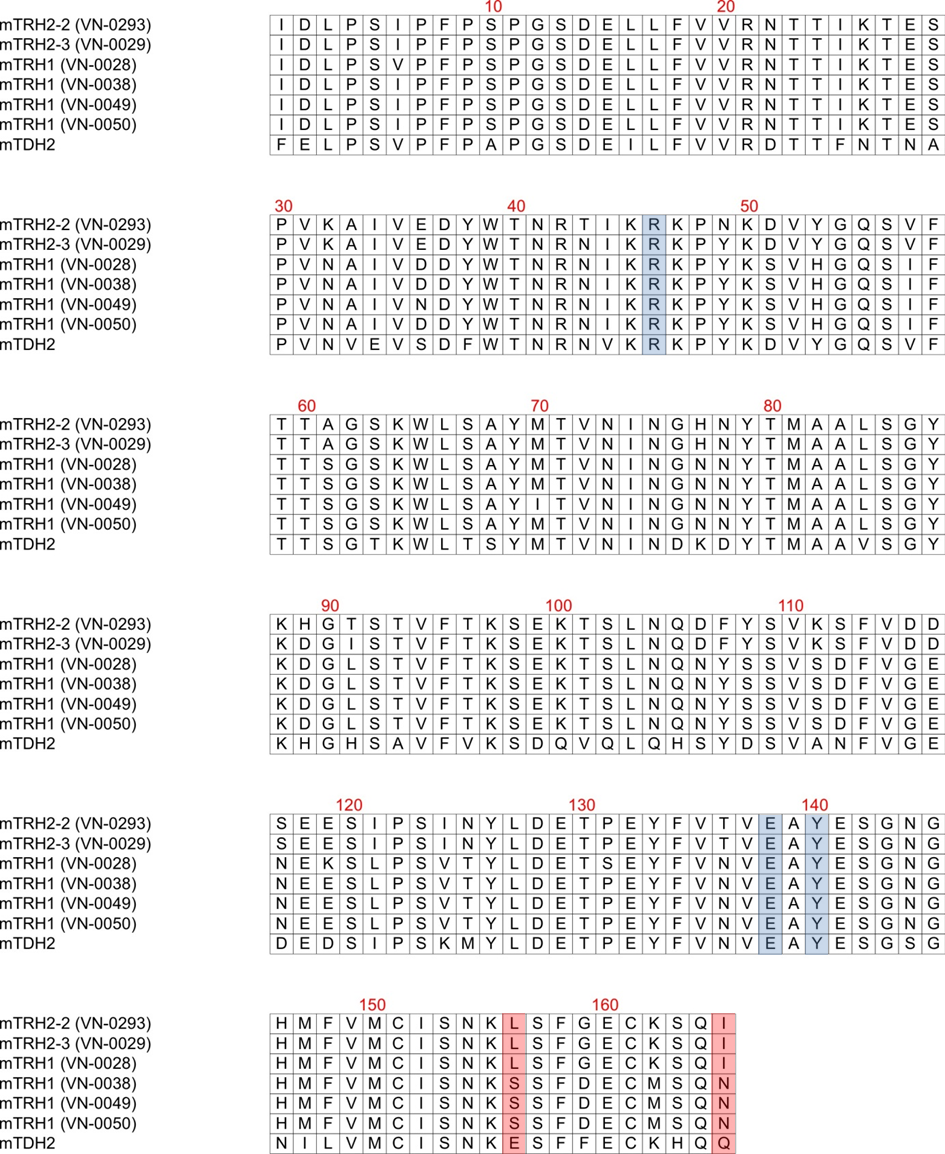

Supplement: S3 Fig — Almost all conserved amino acids described for TDH which may participate in π-cation interactions and maintain tetrameric structures (e.g. R46, E138 and Y140) are conserved in all TRH variants. Two possibly relevant amino acid changes were identified. At position 165 TDH2 and TRH1 (VN-0038) possess a hydrophilic aa residue, glutamine (Q) and asparagine (N) respectively, while TRH1 (VN-0028) and both TRH2 variants possess an isoleucine (I) which is regarded as very hydrophobic. At position 156 TDH2 and TRH1 (VN-0038) exhibit hydrophilic/neutral amino acids (glutamic acid, E; serine, S) while TRH1 (VN-0028) and both TRH2 variants again possess a very hydrophobic amino acid (leucine, L). Using SWISS Model server (http://swissmodel.expasy.org/interactive) predicted 3D structure of TRH variants was modelled on the basis of crystal structure of TDH2—which forms a tetramer in solution as TRH [61, 62]. Amino acids at position 165 and 156 of TDH2 are located within and on the edge of the pore formed by the tetramer. (TIF) [file pone.0118559.s003.tif]
