# Supplementary material for: Characterization of trh2 Harbouring Vibrio parahaemolyticus Strains Isolated in Germany
Source: PLoS One. 2015 Mar 23;10(3):e0118559. doi: 10.1371/journal.pone.0118559 (PMC4370738; doi:10.1371/journal.pone.0118559)
Supplement: S1 Table — Bold letters indicate gene specific sequences. Amplified genes have product size of 533 bp. B) PCR conditions for E-PCR1. PCR reactions were carried out in a 50 μl volume as described [31]. (DOCX) [file pone.0118559.s004.docx]

**Table S1A Primer used for E-PCR1.** Bold letters indicate gene specific sequences. Amplified genes have product size of 533 bp.

| Amplified gene | Primer | Primersequence 5’ → 3’ |
| --- | --- | --- |
| mTRH1 (VN-0028) | X-Vpara-mTRH1(VN-28)-F | AGA AGG AGA TAA ACA – ATG – **ATT GAC CTA CCA TCC GTA CCT** |
|  | X-Vpara-mTRH1(VN-28)-R | C TTG GTT AGT TAG TTA – **TTA AAT TTG TGA TTT ACA TTC GCC A** |
| mTRH1 (VN-0038) | X-Vpara-mTRH1(VN-38)-F | AGA AGG AGA TAA ACA – ATG – **ATT GAC CTG CCA TCC ATA CCT** |
|  | X-Vpara-mTRH1(VN-38)-R | C TTG GTT AGT TAG TTA – **TTA ATT TTG TGA CAT ACA TTC ATC A** |
| mTRH2-3 (VN-0029) | X-Vpara-mTRH2-F | AGA AGG AGA TAA ACA – ATG – **ATT GAT CTG CCA TCA ATA CCT** |
|  | X-Vpara-mTRH2-R | C TTG GTT AGT TAG TTA – **TTA AAT TTG TGA TTT ACA TTC GCC A** |
| mTRH2-2 (VN-00293) | X-Vpara-mTRH2-F | AGA AGG AGA TAA ACA – ATG – **ATT GAT CTG CCA TCA ATA CCT** |
|  | X-Vpara-mTRH2-R | C TTG GTT AGT TAG TTA – **TTA AAT TTG TGA TTT ACA TTC GCC A** |
| mTDH2 (control) | X-Vpara-mTDH-F | AGA AGG AGA TAA ACA – ATG – **TTT GAG CTT CCA TCT GTC CC** |
|  | X-Vpara-mTDH-R | C TTG GTT AGT TAG TTA – **TTA TTG TTG ATG TTT ACA TTC AA** |

m = mature protein (without signal peptide)

**Table S1B PCR conditions for E-PCR1.** PCR reactions were carried out in a 50 µl volume as described [31].

| PCR step | Temperature | | Time duration |
| --- | --- | --- | --- |
|  | mTRH | mTDH |  |
| Initial denaturation | 95 °C | 95 °C | 10 min |
| Denaturation | 94 °C | 94 °C | 30 s  x 30 |
| Primer annealing | **56 °C** | **54 °C** | 30 s |
| Extension | 72 °C | 72 °C | 45 s |
| Final extension | 72 °C | 72 °C | 5 min |
