# Supplementary material for: Characterization of trh2 Harbouring Vibrio parahaemolyticus Strains Isolated in Germany
Source: PLoS One. 2015 Mar 23;10(3):e0118559. doi: 10.1371/journal.pone.0118559 (PMC4370738; doi:10.1371/journal.pone.0118559)
Supplement: S2 Table — Amplified genes have a product size of 728 bp. PCR conditions are described [31]. (DOCX) [file pone.0118559.s005.docx]

**Table S2 Primer used for E-PCR2**. Amplified genes have a product size of 728 bp. PCR conditions as described [31].

| Primer | Primersequence 5’ → 3’ |
| --- | --- |
| Sense Primer | ATGATATCTCGAGCGGCCGCTAGCTAATACGACTCACTATAGGGAGACCACAAC GGTTTCCCTCTAGAAATAATTTTGTTTAACTTTAAGAAGGAGATAAACA |
| Antisense Primer | ATGATATCACCGGTGAATTCGGATCCAAAAAACCCCTCAAGACCCGTTTAGA GGCCCCAAGGGGTACAGATCTTGGTTAGTTAGTTA**TTA** |
