# Supplementary material for: Characterization of trh2 Harbouring Vibrio parahaemolyticus Strains Isolated in Germany
Source: PLoS One. 2015 Mar 23;10(3):e0118559. doi: 10.1371/journal.pone.0118559 (PMC4370738; doi:10.1371/journal.pone.0118559)
Supplement: S3 Table — (DOCX) [file pone.0118559.s006.docx]

**Table S3 Sequence types (STs) with allelic profiles of MLST analysis.**

| Strain | **ST** | **dnaE** | **gyrB** | **recA** | **dtdS** | **pntA** | **pyrC** | **tnaA** |
| --- | --- | --- | --- | --- | --- | --- | --- | --- |
| VN-0396 | **1** | 5 | 52 | 27 | 13 | 17 | 25 | 10 |
| RIMD2210633 | **3** | 3 | 4 | 19 | 4 | 29 | 4 | 22 |
| VN-0394* | **6** | 4 | 13 | 11 | 38 | 18 | 9 | 23 |
| VN-0057 | **26** | 17 | 19 | 13 | 36 | 31 | 32 | 26 |
| VN-0058 |  |  |  |  |  |  |  |  |
| VN-0046 | **34** | 20 | 25 | 15 | 13 | 7 | 11 | 5 |
| VN-0055 | **35** | 20 | 7 | 15 | 13 | 7 | 11 | 5 |
| VN-0045 | **36** | 21 | 15 | 1 | 23 | 23 | 21 | 16 |
| VN-0024 | **50** | 29 | 5 | 22 | 12 | 20 | 22 | 25 |
| VN-0038 | **64** | 40 | 39 | 3 | 18 | 4 | 36 | 29 |
| VN-0061 |  |  |  |  |  |  |  |  |
| VN-0029* | **73** | 3 | 44 | 42 | 44 | 38 | 44 | 24 |
| VN-0030* |  |  |  |  |  |  |  |  |
| VN-0393* |  |  |  |  |  |  |  |  |
| VN-0395* |  |  |  |  |  |  |  |  |
| VN-3933* |  |  |  |  |  |  |  |  |
| VN-3859 |  |  |  |  |  |  |  |  |
| VN-0293* | **79** | 35 | 43 | 38 | 21 | 31 | 35 | 37 |
| VN-2897* |  |  |  |  |  |  |  |  |
| VN-5189* |  |  |  |  |  |  |  |  |
| VN-0050 | **83** | 5 | 52 | 27 | 13 | 17 | 25 | 40 |
| VN-0049 | **91** | 35 | 4 | 16 | 50 | 29 | 5 | 42 |
| VN-10300 | **761** | 47 | 52 | 19 | 287 | 24 | 43 | 37 |
| VN-0028 | **966** | 12 | 312 | 25 | 342 | 26 | 37 | 132 |
| VN-0053 | **967** | 93 | 375 | 71 | 29 | 4 | 45 | 17 |
| VN-0084 | **985** | 28 | 106 | 82 | 251 | 18 | 38 | 2 |
| VN-4016 | **987** | 196 | 380 | 19 | 29 | 193 | 176 | 61 |

uk = unknown
